# Bioorthogonal pro-metabolites for profiling short chain fatty acylation†
†Electronic supplementary information (ESI) available. See DOI: 10.1039/c7sc00247e


**DOI:** 10.1039/c7sc00247e

**Published:** 2017-12-08

**Authors:** Wilson R. Sinclair, Jonathan H. Shrimp, Thomas T. Zengeya, Rhushikesh A. Kulkarni, Julie M. Garlick, Hans Luecke, Andrew J. Worth, Ian A. Blair, Nathaniel W. Snyder, Jordan L. Meier

**Affiliations:** a Chemical Biology Laboratory , Center for Cancer Research , National Cancer Institute , National Institutes of Health , Frederick , MD 21702 , USA . Email: jordan.meier@nih.gov; b National Institute of Diabetes and Digestive and Kidney Diseases , National Institutes of Health , Bethesda , MD 20817 , USA; c Penn SRP Center , Center for Excellence in Environmental Toxicology , University of Pennsylvania , Philadelphia , PA 19104 , USA; d Drexel University , A.J. Drexel Autism Institute , 3020 Market St , Philadelphia , PA 19104 , USA

## Abstract

Short chain fatty acids (SCFAs) play a central role in health and disease. One function of these signaling molecules is to serve as precursors for short chain fatty acylation, a class of metabolically-derived posttranslational modifications (PTMs) that are established by lysine acetyltransferases (KATs) and lysine deacetylases (KDACs). *Via* this mechanism, short chain fatty acylation serves as an integrated reporter of metabolism as well as KAT and KDAC activity, and has the potential to illuminate the role of these processes in disease. However, few methods to study short chain fatty acylation exist. Here we report a bioorthogonal pro-metabolite strategy for profiling short chain fatty acylation in living cells. Inspired by the dietary component tributyrin, we synthesized a panel of ester-caged bioorthogonal short chain fatty acids. Cellular evaluation of these agents led to the discovery of an azido-ester that is metabolized to its cognate acyl-coenzyme A (CoA) and affords robust protein labeling profiles. We comprehensively characterize the metabolic dependence, toxicity, and histone deacetylase (HDAC) inhibitor sensitivity of these bioorthogonal pro-metabolites, and apply an optimized probe to identify novel candidate protein targets of short chain fatty acids in cells. Our studies showcase the utility of bioorthogonal pro-metabolites for unbiased profiling of cellular protein acylation, and suggest new approaches for studying the signaling functions of SCFAs in differentiation and disease.

## Abstract 2

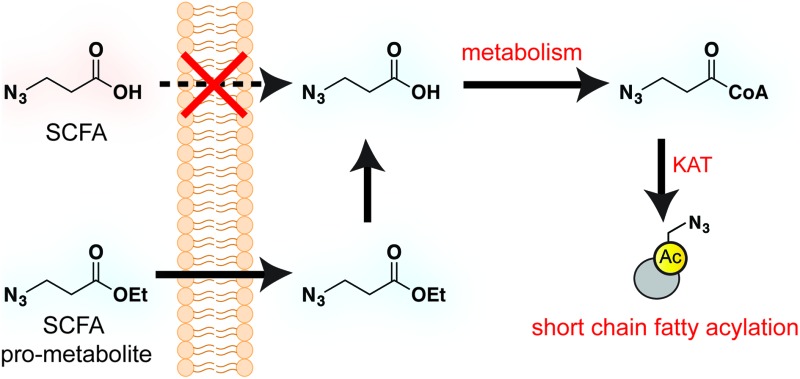
A systematically designed panel of biorthogonal pro-metabolites was synthesized and evaluated as agents for tracing cellular short chain fatty acylation.

## Introduction

**Fig. 1:**
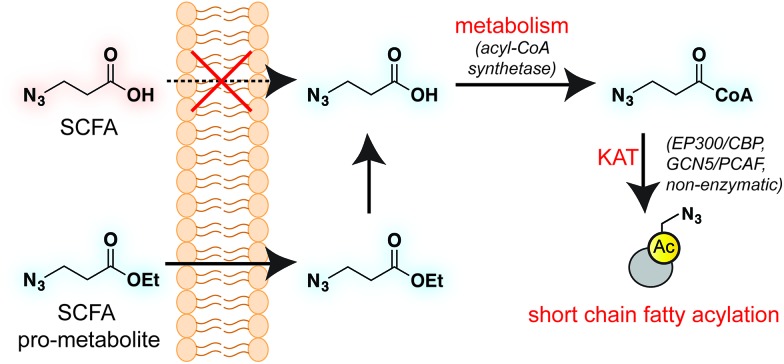
Bioorthogonal pro-metabolite strategy for profiling short chain fatty acylation.

Short chain fatty acids (SCFAs) play a critical role as mediators of health and disease.^1^–^3^ For example, butyrate stimulates differentiation in many cellular models of cancer^4^,^5^ and displays context-dependent effects on tumorigenesis^6^–^8^ and immune cell function^9^,^10^ in whole organisms. In addition to their role in acetyl-coenzyme A (CoA) biosynthesis *via* the β-oxidation pathway and activity as histone deacetylase (HDAC) inhibitors,^11^–^13^ SCFAs also constitute the requisite precursor for protein short chain fatty acylation. This family of posttranslational modifications (PTMs) derives from the metabolism of SCFAs to acyl-CoAs, which may then be used as cofactors by lysine acetyltransferase (KAT) enzymes (Fig. 1).^14^–^16^ Due to its unique reliance on both metabolism and KAT activity, studies of this PTM have significant potential to provide insights into the role of these pathways in disease. Indeed, recent studies of short chain fatty acylation in histones have found these PTMs are dynamic,^17^ can nucleate or impede unique protein–protein interactions,^18^–^20^ and are associated with distinct effects on transcription relative to acetylation.^21^ However, a limitation to our understanding of this process is the fact that relatively few methods for studying short chain fatty acylation exist. Antibody-based analyses of these modifications are limited by the poor affinity and selectivity of antibodies generated against marks such as butyryl- and propionyl-lysine. This may be why these approaches have, to date, identified only a handful of short chain fatty acylated targets outside of histones.^22^–^24^ To address the limitations of antibody-based methods, bioorthogonal metabolic tracing has emerged as a complementary strategy to profile PTMs in living cells.^25^–^32^ The application of this approach to study SCFA-derived acylation was pioneered by Hang and coworkers, who demonstrated that alkynyl-SCFAs could be used to introduce a latent affinity handle into SCFA-modified proteins, enabling their facile visualization and enrichment.^33^ A variant of this approach has also been usefully applied to study lysine malonylation.^34^ However, a challenge of this strategy is that bioorthogonal SCFA tracers must compete with endogenous SCFAs for metabolism and KAT utilization, potentially limiting their efficacy. Thus, the development of highly sensitive bioorthogonal reporters for profiling short chain fatty acylation remains an important goal.

A validated strategy to increase the cellular delivery of small molecules and metabolic tracers is through reversible masking of polar functional groups.^26^,^27^,^35^,^36^ Regarding SCFAs specifically, a number of groups have found butyrate's biological effects to be increased upon masking its polar carboxylate moiety with an ester.^37^,^38^ In analogy with pro-drugs, we refer to such molecules as “pro-metabolites.”^39^ A prototypical SCFA pro-metabolite is tributyrin, a naturally-occurring butyrate ester that displays potent antineoplastic and chemopreventative activity.^40^ Inspired by tributyrin and other pro-metabolites, we hypothesized that masking the polar carboxylate of bioorthogonal SCFAs may similarly increase their potency. Such optimized agents have the potential to provide a robust reporter of short chain fatty acylation, and by proxy, metabolism and cellular KAT/KDAC activity. Towards the goal of defining acylation-dependent signaling in cancer, here we report a bioorthogonal pro-metabolite strategy for profiling short chain fatty acylation (Fig. 1). First, we systematically define the influence of SCFA-ester linkage and bioorthogonal chemotype on cellular protein labeling. Next, we demonstrate the ability of optimized agents to form bioorthogonal acyl-CoAs in cells and establish their cell-type and metabolism-specific labeling properties. Finally, we demonstrate the utility of bioorthogonal pro-metabolites to identify novel targets of SCFAs in cells. These studies highlight the power of pro-metabolite approaches to expand the scope of bioorthogonal metabolic tracing methods, and suggest novel strategies for studying the signaling role of short chain fatty acids in health and disease.

## Results and discussion

### Design of a bioorthogonal pro-metabolite library

**Fig. 2:**
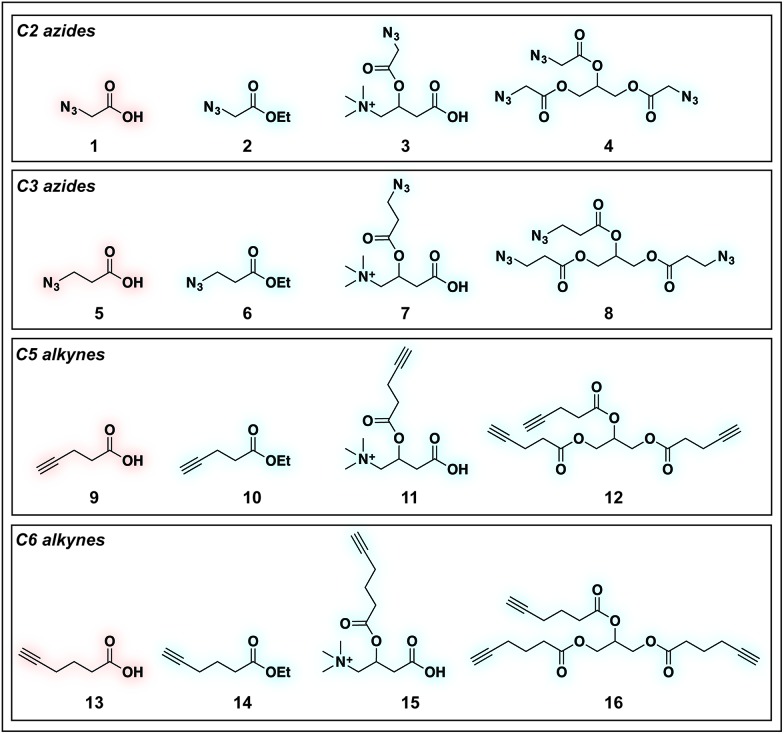
Panel of SCFA pro-metabolites synthesized and evaluated in this study.

To identify sensitive agents for profiling short chain fatty acylation, we focused on exploring three structural elements of bioorthogonal SCFAs (Fig. 2). First we varied the bioorthogonal reporter, reasoning azides (**1–8**) and alkynes (**9–16**) may demonstrate differential metabolic processing and detection sensitivities. Second, we varied the length of the alkyl chain between the SCFA carboxylate and bioorthogonal reporter. Previous studies have found SCFA chain length to be a crucial factor in the formation of SCFA-CoAs by acyl-CoA synthetases,^41^–^43^ and the utilization of these SCFA-CoAs by KATs.^15^,^44^ Third, we varied the carboxylate ester moiety. Here we focused on simple ethyl esters (**2**, **6**, **10**, **14**), as well as carnitine and triacylglycerol esters (Fig. 2). Carnitine esters (**3**, **7**, **11**, **15**) were designed to facilitate delivery of SCFAs to the mitochondria, an organelle that shows robust acyl-CoA metabolism.^45^ In contrast, tributyrin-inspired triacylglycerol esters (**4**, **8**, **12**, **16**) were designed to take advantage of the known increased cellular uptake of highly lipophilic molecules, as well as the ability of these esters to be specifically cleaved by endogenous triacylglycerol lipases. An additional advantage of these molecules relative to other esters is their ability to enable suprastoichiometric delivery (three equivalents) of their cognate bioorthogonal SCFAs. This panel of pro-metabolites was readily synthesized using straightforward ester bond formation from the parent bioorthogonal carboxylates (Scheme S1†). While the majority of bioorthogonal SCFA derivatives were obtained in good yield, we found carnitine esters to be the most difficult targets due to their challenging purification. Similar optimization was required to obtain triacylglycerol SCFAs, as literature conditions yielded a complex mixture of mono-, di-, and triacylglycerides. These studies establish a systematic panel of bioorthogonal SCFAs to define the structure–activity relationship of pro-metabolites in living cells.

### Evaluation of bioorthogonal pro-metabolites for *in vitro* protein labeling

**Fig. 3:**
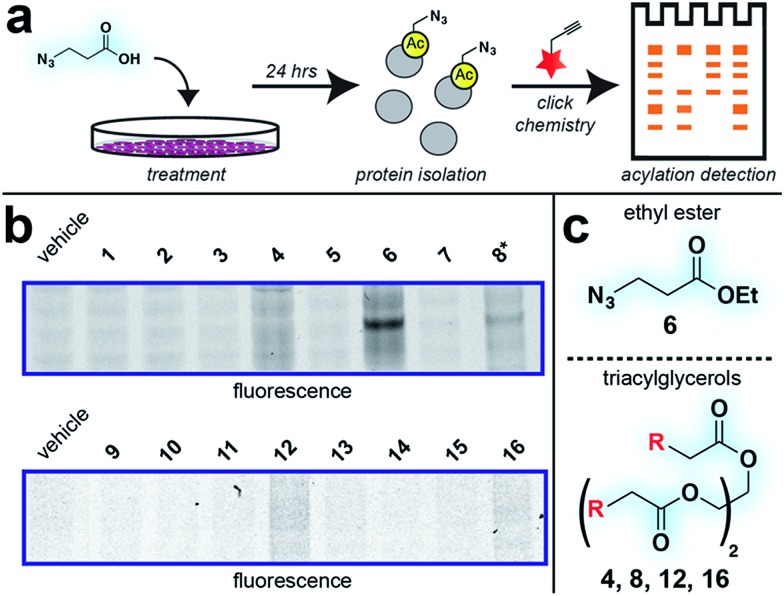
Cellular evaluation of bioorthogonal SCFA pro-metabolites. (a) Scheme for cellular analysis and assay of pro-metabolite incorporation *via* fluorescent gel-based assay. (b) Protein labeling by pro-metabolites and parent carboxylates in HepG2 cells (2.5 mM, 24 h). Asterisk indicates a pro-metabolite assessed at 1 mM due to toxicity. Lane 1 of each gel was cropped from its original position. Full gel images and Coomassie loading controls are provided in the ESI.† (c) Most active bioorthogonal SCFA pro-metabolite scaffolds.

With these compounds in hand, we next evaluated our library for protein labeling in HepG2 human hepatocellular carcinoma cells (Fig. 3). Our choice of liver cancer as a model for evaluation of our pro-metabolites stems from the fact that the liver is a major site of SCFA metabolism *in vivo*, where it utilizes abundant acyl-CoA synthetase activities to transform SCFAs into acyl-CoAs that support short chain fatty acylation.^46^ Accordingly, we treated HepG2 cells with each of our pro-metabolites at a single concentration for 24 hours. Cells lysates were harvested, subjected to click chemistry with a fluorescent azide or alkyne, and analyzed by SDS-PAGE (Fig. 3a). Consistent with previous reports, detection of azide-labeled proteins using a fluorophore alkyne manifested uniformly higher background labeling.^47^ Pro-metabolites demonstrating the most intense protein labeling profiles were the bioorthogonal triacylglycerides (**4**, **8**, **12**, **16**) as well as ethyl azidopropionate (**6**) (Fig. 3b and c). Labeling by triacylglycerides **8** and **12** was accompanied by noticeable toxicity at 24 hours (Fig. S1a†). Interestingly, analysis of the bioorthogonal alkyne series revealed pentynoate esters to be consistently more toxic than their hexynoate counterparts (Fig. S1b†). This did not correlate with protein labeling or HDAC inhibitory activity (Fig. 3 and S2†), suggesting pentynoate may manifest its toxic affects through alternative mechanisms. Of note, 5-pentynoic acid structurally resembles 5-pentenoic acid, a cytotoxic agent known to form electrophilic species and inhibit fatty acid oxidation in cells.^48^ This toxicity (as well as protocol-specific variability *e.g.* dose, cell line choice) may explain the modest labeling by **9**, which has been validated as a biorthogonal protein labeling reagent by multiple groups.^33^,^49^ Analysis of the active azide pro-metabolites (**4**, **6**, **8**) caused us to focus on **6** as a lead pro-metabolite. Key rationale for this choice included the fact that **6** provides the most intense labeling of any bioorthogonal SCFA precursor examined (Fig. 2b), and robustly labels proteins at concentrations that do not impede cell growth (Fig. S1c and d†). Acid extraction and LC-MS metabolomics analysis of cells treated with **6** confirmed formation of azidopropionyl-CoA, consistent with the ability of **6** to form cofactors for protein short chain fatty acylation reactions (Fig. S3†). To our knowledge this represents first direct evidence of bioorthogonal acyl-CoA formation in living cells. These data, combined its facile synthesis, prompted us to further evaluate **6** as an optimized reporter of cellular short chain fatty acylation.

### Mechanistic analysis of a lead pro-metabolite for short chain fatty acylation profiling

**Fig. 4:**
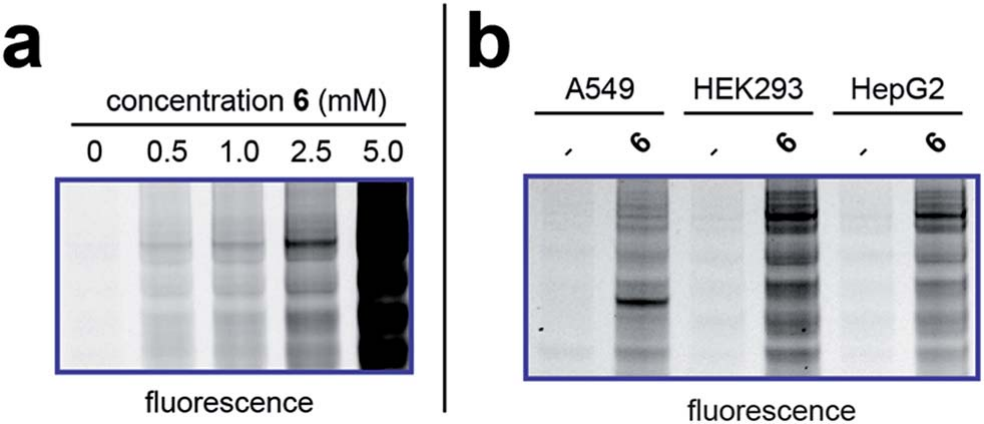
Cellular evaluation of lead pro-metabolite **6**. (a) Dose-dependent labeling of HepG2 cells. Cells were treated with **6** for 6 h. (b) Cell-line specific protein labeling by pro-metabolite **6**. Full gel images and Coomassie loading controls are provided in the ESI.†

To better define the activity of pro-metabolite **6**, we next evaluated it in a series of experiments using our fluorescent gel-based assay. Cellular labeling of proteins by **6** was dose-dependent, with significant labeling observed at concentrations as low as 0.5 mM (Fig. 4a). Analyzing the time-dependent activity of **6** found labeling to peak between 6 and 9 hours (Fig. S1d†). Of note, the reduced labeling observed at 24 hours suggests short chain fatty acylation implemented by **6** may be reversible, consistent with the known ability of many HDAC enzymes to deacetylate fatty acyl-lysine derivatives.^50^,^51^ To assess the generality of pro-metabolite **6**, we compared cell labeling in HepG2 to labeling in cell lines derived from a variety of tissues of origin, including lung (A549) and kidney (HEK293). Interestingly, each cell line demonstrated intense, but unique, patterns of protein labeling by **6** (Fig. 4b). This suggests the ability of **6** to function as a useful reporter of short chain fatty acylation in a number of tissues and cell types.

**Fig. 5:**
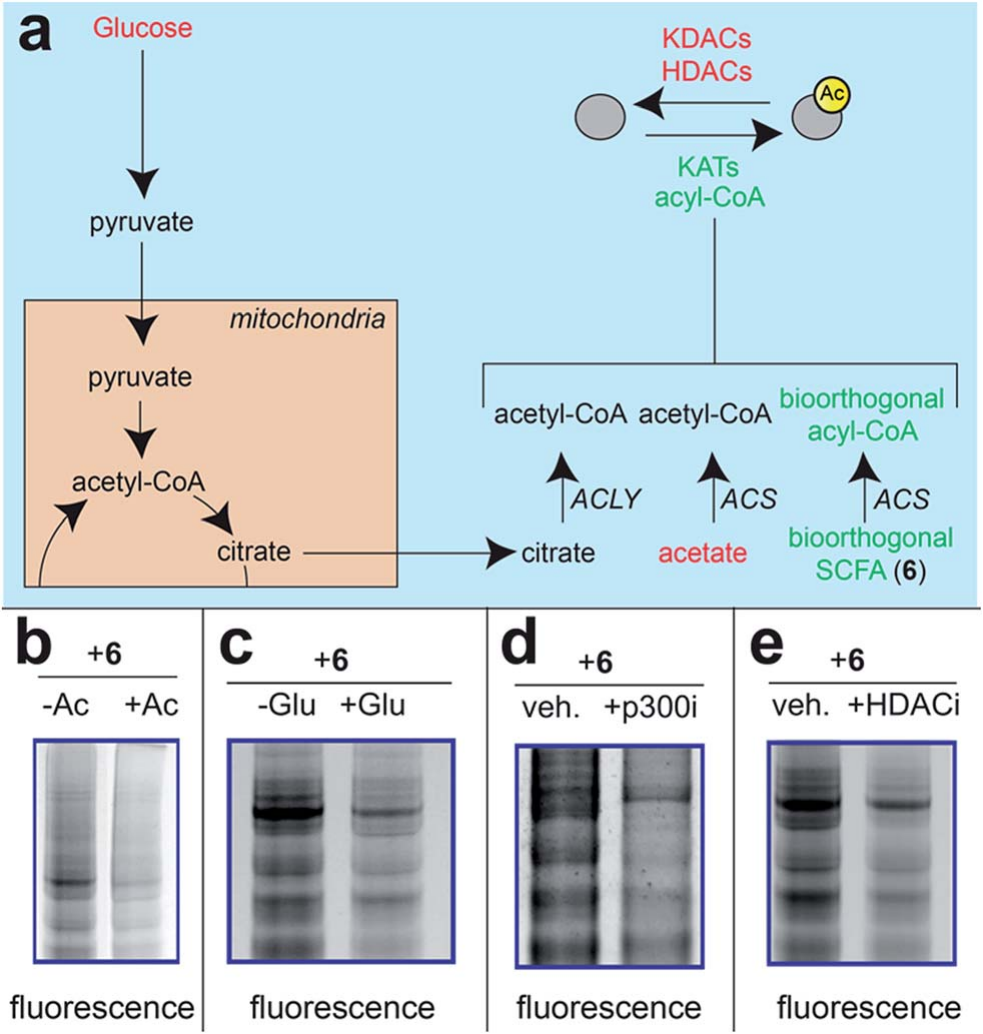
Pro-metabolites are metabolism and HDAC-dependent labeling agents. (a) Schematic depicting biosynthetic origins of acetyl-CoA and SCFA-CoAs used in lysine acylation reactions. Agents in red are manipulated in these studies. ACS = acyl-CoA synthetase enzymes. (b) Protein labeling by bioorthogonal reporter **6** is competed by exogenous SCFAs. (c) Protein labeling by bioorthogonal reporter **6** is enhanced in the absence of glucose, which favors ACS activity. (d)Protein labeling by bioorthogonal reporter **6** is decreased by pretreatment of cells with a spirocyclic p300 inhibitor. (e) Protein labeling by bioorthogonal reporter **6** is decreased by pretreatment of cells with the HDAC inhibitor SAHA. All experiments were performed in HepG2 cells, except for KATi treatment which was performed in HEK293. Full gel images and Coomassie loading controls are provided in the ESI.†

Next, we sought to better understand the metabolic determinants of short chain fatty acylation by **6** (Fig. 5). First, we explored whether labeling of proteins by **6** was sensitive to competition by endogenous short chain fatty acids such as acetate. Our hypothesis was that acetate may compete with **6** for acyl-CoA synthetase active sites,^46^ thereby reducing bioorthogonal acyl-CoA biosynthesis and competing for sites of protein labeling (Fig. 5a). Consistent with this hypothesis, we observed decreased labeling of cellular proteins by **6** upon co-administration of acetate (Fig. 5b). Treatment of cells with fatty acid synthase (FASN) inhibitors did not substantially reduce labeling by **6** (Fig. S4†), indicating that metabolism of azidopropionate to long chain fatty acyl-CoAs (such as those used as palmitoyltransferases) is not a major mechanism of labeling. Next, we tested the effect of media glucose on labeling on short chain fatty acylation by **6**. Glucose-derived acetyl-CoA may compete with SCFA-CoAs for protein acetylation. In addition, previous studies have found that limiting glucose causes increased utilization of SCFAs for energy production *via* fatty acid oxidation.^6^ Importantly, this bioenergetic shift requires increased biosynthesis of SCFA-CoAs, and should therefore increase labeling by **6** (Fig. 5a) indeed, we observed increased labeling of cellular proteins by **6** when cells were grown in low glucose compared to nutrient replete media (Fig. 5c). Finally, we explored the impact of acetylation dynamics on metabolic labeling. To probe the role of KATs in short chain fatty acylation, we applied a spirocyclic EP300 inhibitor scaffold recently reported in the literature (p300i; Fig. S5a†).^52^,^53^ Inhibition of EP300 reduced the acylation of some, but not all, proteins labeled by **6** (Fig. 5d). This suggests EP300 may collaborate with other enzymatic^21^ and non-enzymatic^54^ mechanisms to establish short chain fatty acylation. Due to the lack of well-validated chemical probes for other KAT enzymes,^55^,^56^ we next assessed how labeling of proteins by **6** was impacted by HDAC inhibitors. Treatment of cells with the HDAC inhibitor SAHA was found to upregulate global acetylation, while coordinately decreasing labeling of cellular proteins by **6** (Fig. 5e). This indicates active deacetylation may be required to liberate lysines for short chain fatty acylation by bioorthogonal acyl-CoAs. These studies define pro-metabolite **6** as a general reporter of SCFA metabolism and acylation.

### Profiling the cellular targets of bioorthogonal SCFAs

Finally, we sought to demonstrate the ability of **6** to identify specific targets of short chain fatty acylation in living cells. As a proof-of-concept, we exposed human embryonic kidney cells to either a pulse of **6** or vehicle DMSO for six hours. Following isolation of cell lysates, proteins labeled by **6** were chemoselectively ligated to a biotin alkyne *via* Cu-catalyzed [3 + 2] cycloaddition, and enriched over streptavidin-agarose. Enriched proteins were subjected to an on-bead tryptic digest and identified by LC-MS/MS. Candidate short chain fatty acylation targets were defined as proteins enriched greater than 2-fold from cells treated with **6**, relative to a vehicle control (Table S1†). Analyzing the abundance of these targets *via* spectral counting identified 447 low abundance proteins (1–5 spectral counts), 121 medium abundance proteins (5–10 spectral counts), and 91 highly abundant proteins (>10 spectral counts). Of note, many of these proteins, including 41% of highly abundant enriched proteins, have previously been identified as targets of short chain fatty acylation by Hang and coworkers (Table S1†).^33^ Gene ontology analysis also supported the utility of **6** as a bioorthogonal reporter, with acetylation being the most strongly enriched term (*P* = 1.5 × 10^–153^) (Table S2†). This analysis also revealed targets of **6** were enriched in nuclear proteins, consistent with the nuclear localization of many KATs (Table S3†). Of note, among proteins identified in triplicate datasets was EP300, which has previously been observed to catalyze short chain fatty acylation. EP300 may be a target of this modification *via* autoacetylation,^22^,^57^–^60^ and follow-up studies validated the ability of **6** to label ectopically-expressed EP300 (Fig. S5b and c†). While we defer a more detailed validation and discussion of these candidate SCFA targets for future studies, overall these findings support the ability of **6** to be applied in global proteomic studies of short chain fatty acylation.

## Conclusions

Here we have described a bioorthogonal pro-metabolite strategy for profiling cellular short chain fatty acylation. Specifically, we find that several alkyne- and azide-SCFAs function as effective protein labeling reagents when delivered as triglyceride analogues (**4**, **8**, **12**, **16**), or as ethyl esters (**6**). Detailed characterization of lead pro-metabolite **6** found that it can form bioorthogonal acyl-CoAs in cells, is active in several cell models, and labels proteins in a manner reflective of cellular metabolism and acetylation dynamics. Our studies suggest that pro-metabolite strategies, which have previously been used to optimize incorporation of azide- and alkyne-containing glycans,^26^,^36^ may have broad utility in the delivery of bioorthogonal metabolic tracers. It is important to highlight that our work does not stand alone, but is connected to that of other groups that have used acetoxymethyl esters,^34^ as well as O- and N-linked hexoasamines to deliver SCFAs to cells.^36^,^49^ The ability of these scaffolds to deliver biorthogonal SCFAs has not yet been systematically determined, and in the future it may be informative to compare them to the ethyl esters and triacylglycerols investigated here. Looking forward, we anticipate several applications for the potent SCFA pro-metabolites identified in this study. First, the strong labeling afforded by **6** suggest it may be a useful *in vivo* tracer for identifying endogenously short chain fatty acylated proteins associated with SCFA-driven phenotypes such as differentiation, anti-inflammation, or tumor suppression.^4^,^6^–^9^ To be most impactful, such studies will require that pro-metabolites recapitulate the phenotypic effects of endogenous SCFAs. As many of these effects have been associated with the HDAC activity of SCFAs, it is promising that our cellular studies indicate the azido- and alkynyl-SCFAs investigated here also stimulate histone acetylation (Fig. S2†). Second, bioorthogonal pro-metabolites may provide a facile readout of global KAT/KDAC activity in cellular or *in vivo* systems, complementing current chemical proteomic approaches, which are only applicable in cell lysates.^61^,^62^ Finally, bioorthogonal SCFA pro-metabolites may facilitate chemical genetic approaches to identify specific KAT substrates in cells.^63^ Of note, mutations have been reported that confer KAT2A (GCN5L2) and KAT8 (MOZ) with the ability to use elongated azido- and alkynyl-CoAs.^64^ Overall, these studies represent a key methodological advance in the development of new methods to understand acetylation-dependent signaling in living systems.

## Conflicts of interest

The authors declare that there is no conflict of interest regarding the publication of this article.

## Supplementary Material

Supplementary information

Supplementary information
