# Supplementary material for: Bioorthogonal pro-metabolites for profiling short chain fatty acylation
Source: Chem Sci. 2017 Dec 8;9(5):1236–41. doi: 10.1039/c7sc00247e (PMC5885804; doi:10.1039/c7sc00247e)
Supplement: Supplementary file 2 [file SC-009-C7SC00247E-s002.pdf]

Electronic Supplementary Material (ESI) for Chemical Science.
This journal is © The Royal Society of Chemistry 2017

Supporting Information

Bioorthogonal pro-metabolites for profiling short chain fatty acylation

*Wilson R. Sinclair, Jonathan H. Shrimp, Thomas T. Zengeya, Rhushikesh A. Kulkarni, Julie M. Garlick, Hans Luecke, Andrew J. Worth, Ian A. Blair, Nathaniel W. Snyder, and Jordan L. Meier**

Chemical Biology Laboratory, National Cancer Institute, Frederick, MD, 21702

Table of Contents for Supporting Information

	Page
Supplemental Figures S1-S5	S2-7
General synthetic procedures and materials	S8
Synthesis and characterization data for pro-metabolites	S9-17
^{1}H- and ^{13}C-NMR spectra for pro-metabolites	S18-26
General procedures and materials for cellular assays	S27-28
Treatment of cells with pro-metabolites for metabolic labeling analysis	S28
Mass spectrometry characterization of azidopropionyl-CoA formation	S29
Uncropped gels, immunoblots, and loading controls	S30-50
References	S51

Supplemental Figures

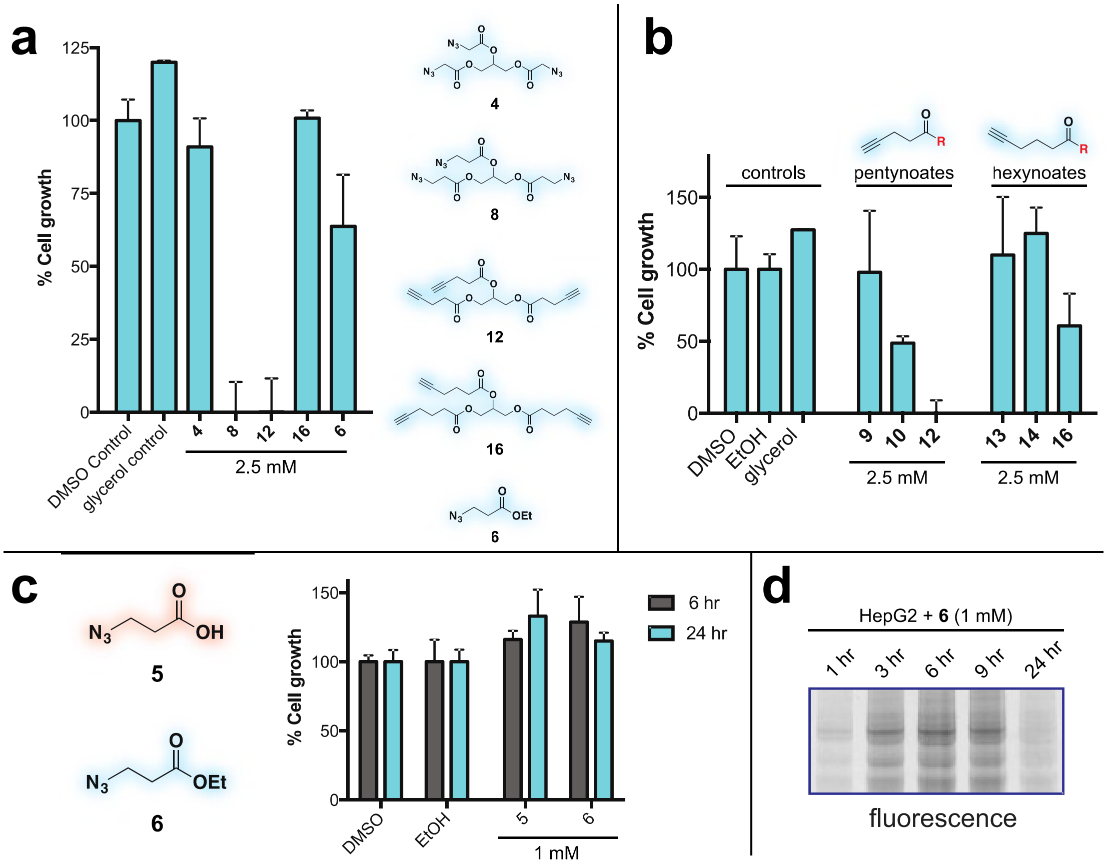

Figure S1. (a) Toxicity of active pro-metabolites at 24 h in HepG2 cells. (b) Relative toxicity of pentynoate and hexynoate analogues at 48 h in HepG2 cells. (c&d) Pro-metabolite **6** can be applied under conditions (1 mM, 6 h) that minimize cell death and enable maximal protein labeling in HepG2 cells.

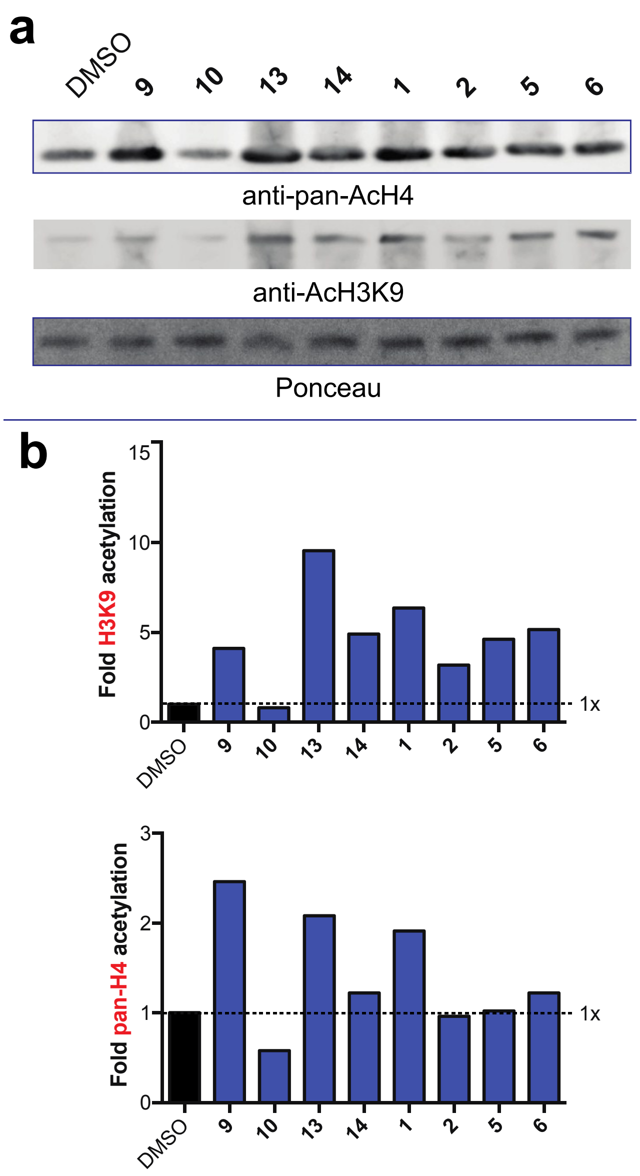

Figure S2. HDAC inhibitory activity of ethyl ester pro-metabolites and free acids. (a) Western blots of histone acetylation in HEK293 cells following 6 h treatment with bioorthogonal acids (**1**, **5**, **9**, **13**) or bioorthogonal esters (**2**, **6**, **10**, **14**) at 2.5 mM. (b) Bar chart illustration depicting observed histone acetylation changes.

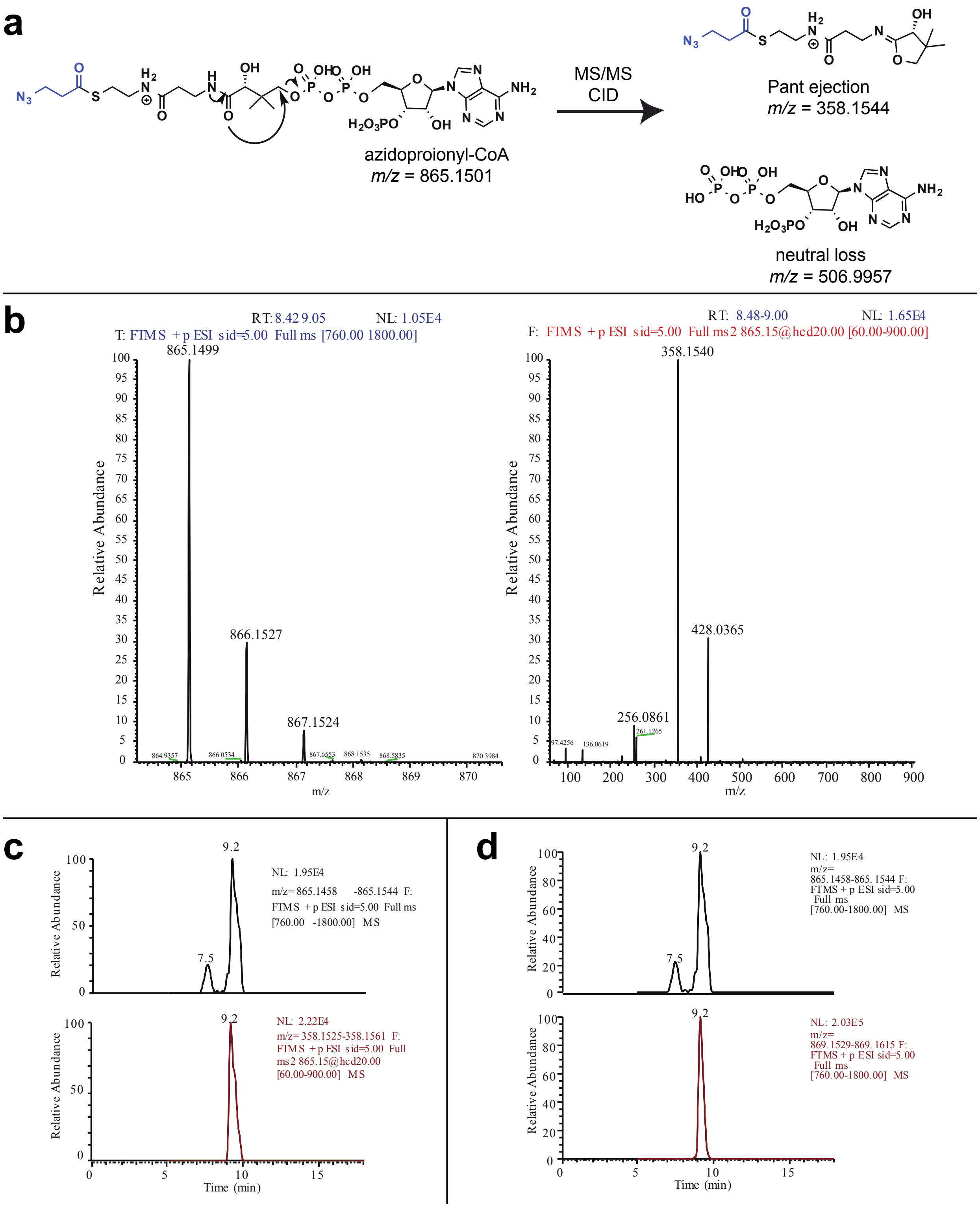

Figure S3. | see next page for caption

LC-MS/MS analysis of azidopropionyl-CoA formation. (a) Product ions derived from acyl-CoA analogues through collision-induced dissociation and MS/MS analysis. (b) HRMS (left) and MS/MS (right) of synthetically prepared azidopropionyl-CoA. (c) HRMS and LC-MS/MS of Hepa1c1c7 cell extract demonstrating co-eluting fragments specific to azidopropionyl-CoA. Top: 867.15 *m/z* corresponding to 3-azidopropionyl-CoA. Bottom: 358.15 *m/z* corresponding to pantetheine ejection fragment. Cells were treated with 0.1 mM ethyl azidopropionate (**6**) for 1 hr. Analogous results were observed in MCF7 and HepG2 cells treated with **6**. (d) LC-HRMS demonstrating co-elution of azidopropionyl-CoA and $^{13}C_3$ $^{15}N_1$-azidopropionyl-CoA (9.2 min), which are well resolved from a near isobaric interference at 7.5 min.

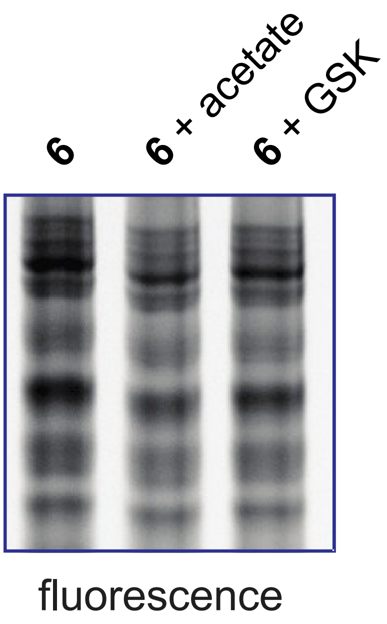

Figure S4. Labeling by pro-metabolite **6** is not FASN-dependent. Cells were treated with pro-metabolite **6** (1 mM, 6 hours) following pre-treatment with FASN inhibitors orlistat (25 μM) or GSK2194069 (100 μM).

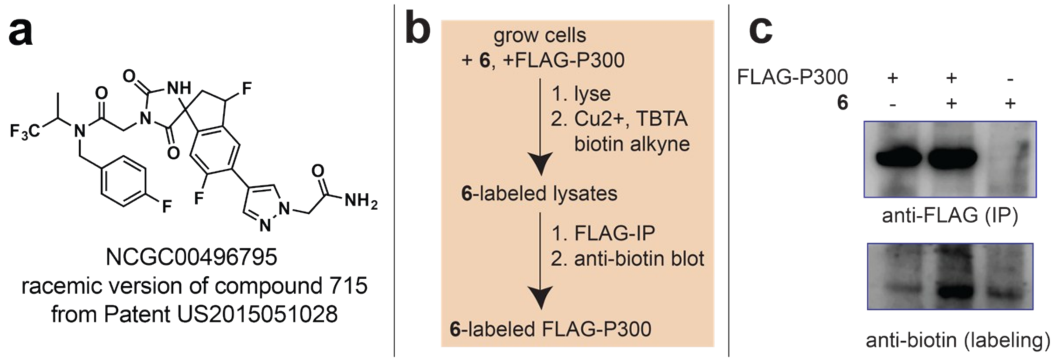

Figure S5. (a) Structure of p300 KAT inhibitor (KATi) from patent literature.[1] (b) Experimental workflow for assessing labeling of overexpressed FLAG-EP300 by pro-metabolite **6**. Cells were transfected with FLAG-EP300 for 48 h, followed by treatment with **6** (5 mM) for 6 h. Cells were lysed, subject to Cu-catalyzed azide-alkyne cycloaddition with biotin alkyne, enriched via FLAG immunoprecipitation kit (Sigma), and analyzed for EP300 capture and **6**-labeling by Western blot. Control experiments were performed in parallel, in which **6** or FLAG-EP300 were omitted, respectively. (c) Assessing capture (anti-FLAG IP, anti-FLAG Western) and labeling (anti-FLAG IP, anti-biotin Western) after treatment with **6** and click chemistry with biotin alkyne.

General synthetic procedures and materials

Chemicals were purchased from commercial sources (Sigma-Aldrich, Alfa Aesar, and TCI America) and used without further purification unless otherwise noted. 2-azidoacetatic acid (**1),** ethyl azidoacetate (**2**), 4-pentynoic acid (**9**), ethyl pentynoate (**10**), hexynoic acid (**13**), and ethyl hexynoate (**14**) were obtained from Sigma Aldrich. NCGC00496795 (racemic KATi) was synthesized by Chemogenics Biopharma.[1] Flash column chromatography was performed using normal phase on a CombiFlash® Rf 200i (Teledyne Isco Inc). ^{1}H NMR spectra were recorded at 400 or 500 MHz, and are reported relative to deuterated solvent signals. Data for ^{1}H NMR spectra are reported as follows: chemical shift (δ ppm), multiplicity, coupling constant (Hz), and integration. ^{13}C NMR spectra were recorded at 100 or 125 MHz. Data for ^{13}C NMR spectra are reported in terms of chemical shift. Analytical LC/MS was performed using a Shimadzu LCMS-2020 Single Quadrupole utilizing a Kinetex 2.6 μm C18 100 Å (2.1 x 50 mm) column obtained from Phenomenex Inc. Runs employed a gradient of 0→90% MeCN/0.1% aqueous formic acid over 4 minutes at a flow rate of 0.2 mL/min. Microwave experiments were carried out using the Biotage Initiator 4.0.1.

Synthesis and characterization data for pro-metabolites

(i) 3-(2-azidoacetoxy)-4-(trimethylammonium) butanoate (3)

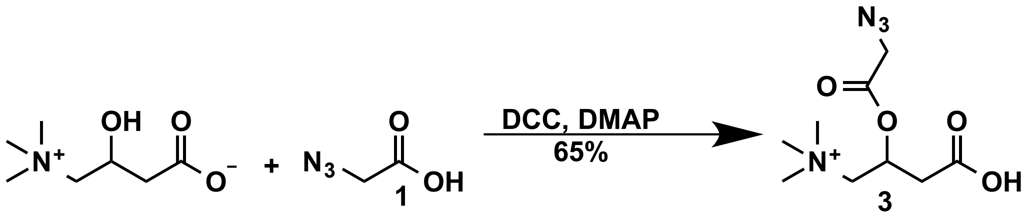

2-azidoacetic acid (**1**) (37μL, 0.5 mmol), carnitine (96.6 mg, 0.6 mmol) and DMAP (5 mg, 0.04 mmol) were dissolved in anhydrous DMF and chilled using an ice bath. *N*,*N*'-dicyclohexylcarbodiimide (DCC) (123 mg, 0.6 mmol) was added and stirred for 10 minutes. The ice bath was removed and the reaction was stirred overnight at room temperature. The reaction mixture was filtered and DMF was removed *in vacuo*. The purified compound was isolated by HPLC using a mobile phase consisting of a gradient of MeCN in 0.1% trifluoroacetic acid (aqueous). HPLC purification was performed using an Agilent 1250 Infinity HPLC equipped with a semi-preparative Phenomenex Gemini C18 column (150 x 21.2 mm, 10 μm) yielding product **3** as an oil (65 mg, 44%). ^{1}H NMR (400 MHz, D_2O) δ 5.68 (q, *J* = 4 Hz, 1H), 3.91 (dd, *J* = 8 Hz, 4Hz 1H), 3.84 (s, 2H), 3.68 (d, *J* = 12 Hz, 1H), 3.16 (s, 9H), 2.82 (m, 2H). ^{13}C NMR (126 MHz, D_2O) δ 174.95, 172.99, 68.68, 65.77, 54.12, 50.80, 36.84. ESI MS: [M+H]+ calculated: 245.26, [M+H]+ found: 246.30.

(ii) propane-1,2,3-triyl tris(2-azidoacetate) (4)

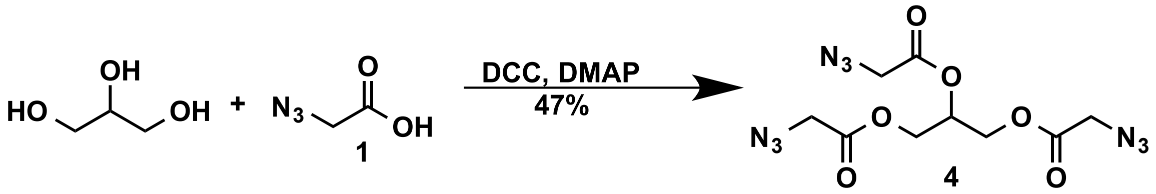

2-azidoacetic acid (**1**) (0.22 mL, 3 mmol), glycerol (92 mg, 1 mmol) and DMAP (5 mg, 0.04 mmol) were dissolved in anhydrous DMF and chilled using an ice bath. DCC (687 mg, 3.33 mmol) was added and stirred for 10 minutes. The ice bath was removed and the reaction was stirred overnight at room temperature. The reaction mixture was filtered and DMF was removed *in vacuo*. The purified compound was isolated using flash chromatography (0→30% dichloromethane: methanol) to yield product **4** as an oil (79 mg, 23%). ^{1}H NMR (500 MHz, $CDCl_3$) δ 5.37 (m, 1H), 4.47 (dd, *J* =10.0 Hz, 5.0 Hz, 2H) 4.26, (dd, *J* =5.0 Hz, 5.0 Hz, 2H), 3.86 (s, 6H). ^{13}C NMR (101 MHz, $CDCl_3$) δ 167.91, 167.67, 70.12, 62.92, 50.22, 50.18. ESI MS: [M+H]+ calculated: 341.24, [M+H]+ found: 342.4.

(iii) 3-azidopropanoic acid (5)

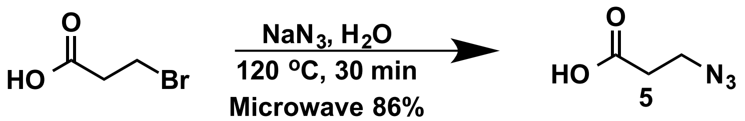

3-bromopropanoic acid (152 mg, 1 mmol), sodium azide (163 mg, 2.5 mmol) were dissolved in 2 mL of water in a 10 mL microwave reaction vessel. The mixture was microwaved at 120 °C for 30 minutes with stirring. The reaction was allowed to cool to room temperature, extracted with diethyl ether, dried over Na_2SO_4, and solvent removed *in vacuo*. The purified compound was isolated and

by flash chromatography (0→30% dichloromethane: methanol) to yield product **5** as a colorless oil (99 mg, 86%). ^{1}H NMR (500 MHz, $CDCl_3$) δ 11.68 (s, 1H), 3.58 (t, *J* =12 Hz, 2H), 2.64 (t, *J* = 12 Hz, 2H). ^{13}C NMR (126 MHz, $CDCl_3$) δ 177.47, 46.36, 33.72. ESI MS: [M+H]+ calculated: 115.09, [M+H]+ found: 116.2.

(iv) ethyl 3-azidopropanoate (6)

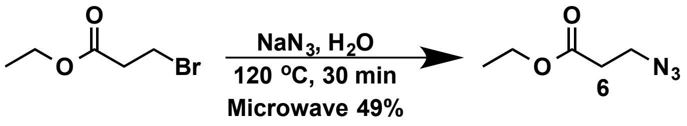

Ethyl-3-bromopropanoic acid (1.28 ml, 10 mmol), sodium azide (1.63 g, 25 mmol) were dissolved in 10 mL of water in a 10-20 mL microwave reaction vessel. The mixture was microwaved at 120 °C for 30 minutes with stirring. The reaction was allowed to cool to room temperature, extracted with diethyl ether, dried over Na_2SO_4, and solvent removed *in vacuo*. The purified compound was isolated and by flash chromatography (0→30% dichloromethane: methanol) to yield product **6** as a colorless oil (704.6 mg, 49%). ^{1}H NMR (400 MHz, $CDCl_3$) δ 4.21 (q, 8 Hz, 2H), 3.60, (t, 8 Hz, 2H), 2.60 (t, 8 Hz, 2H), 1.31 (t, 8 Hz, 3H).
^{13}C NMR (126 MHz, $CDCl_3$) δ 170.81, 60.94, 46.82, 34.03, 14.13. ESI MS:[M+H]+ calculated: 143.15, [M+H]- found: ESI MS: 144.2.

(v) 3-((3-azidopropanoyl)oxy)-4-(trimethylammonio)butanoate (7)

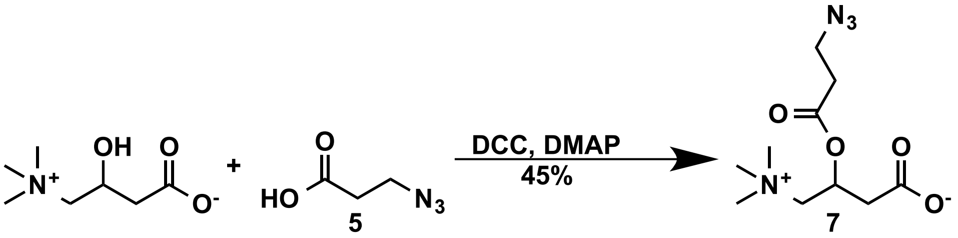

3-azidopropionic acid (**5**) (85 μL, 1 mmol), carnitine (170 mg, 1.1 mmol) and DMAP (5 mg, 0.04 mmol) were dissolved in anhydrous DMF and chilled using an ice bath. DCC (229 mg, 1.1 mmol) was added and stirred for 10 minutes. The ice bath was removed and the reaction was stirred overnight at room temperature. The reaction mixture was filtered and DMF was removed *in vacuo*. The purified compound was isolated by HPLC using a mobile phase consisting of a gradient of MeCN in 0.1% trifluoroacetic acid (aqueous). HPLC purification was performed using an Agilent 1250 Infinity HPLC equipped with a semi-preparative Phenomenex Gemini C18 column (150 x 21.2 mm, 10 μm) to yield product **7** as an oil (116 mg, 45%). ^{1}H NMR (400 MHz, D_2O) δ 5.67 (q, *J* = 4 Hz, 1H), 3.89 (dd, *J* = 8 Hz, 4Hz 1H), 3.65 (t, 8Hz, 2H), 3.59 (d, *J* = 12 Hz, 1H), 3.12 (s, 9H), 2.85 (m, 2H), 2.61 (t, 8 Hz, 2H). ^{13}C NMR (126 MHz, D_2O) δ 174.36, 172.65, 68.40, 65.94, 54.36, 50.75, 46.92, 36.76. ESI MS: [M+H]+ calculated: 259.28, [M+H]+ found: 260.32

(vi) propane-1,2,3-triyl tris(3-azidopropanoate)

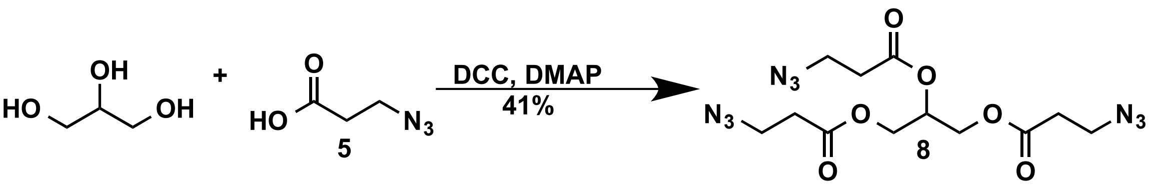

3-azidopropionic acid (**5**) (127.47 mg, 1 mmol), glycerol (33 mg, 0.36 mmol) and DMAP (5 mg, 0.04 mmol) were dissolved in anhydrous DMF and chilled using an ice bath. DCC (229 mg, 1.1 mmol) was added and stirred for 10 minutes. The ice bath was removed and the reaction was stirred overnight at room temperature. The reaction mixture was filtered and DMF was removed *in vacuo*. The purified compound was isolated by flash chromatography (0→30% CH_2Cl_2: MeOH) to yield product **8** as an oil (157 mg, 41%). ^{1}H NMR (400 MHz, $CDCl_3$) δ 5.37 (m, 1H), 4.43 (dd, 8 Hz, 4 Hz, 2H), 4.27 (dd, 4 Hz, 4Hz, 2H), 3.61 (t, 8 Hz, 6H), 2.64 (m, 6H). ^{13}C NMR (101 MHz, $CDCl_3$) δ 170.40, 170.10, 69.46, 62.44, 46.60, 33.88, 33.71. ESI MS: [M+H]+ calculated: 383.33, [M+H]+ found: 383.5.

(vii) ethyl pent-4-ynoate (10)

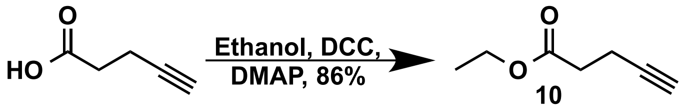

4-pentynoic acid (49 mg, 1 mmol), ethanol (58 μL, 2 mmol) and DMAP (5 mg, 0.04 mmol) were dissolved in anhydrous DMF and chilled using an ice bath. DCC (229 mg, 1.1 mmol) was added and stirred for 10 minutes. The ice bath was removed and the reaction was stirred overnight at room temperature. The reaction mixture was filtered and DMF was removed *in vacuo*. The purified compound was isolated by flash chromatography (0→100% hexane: ethyl acetate) to yield product **10** as an oil (108 mg, 86%). ^{1}H NMR (500 MHz, $CDCl_3$) δ 4.10 (q, *J* = 4 Hz, 2H), 2.48 (m, 4H), 1.91 (t, *J* = 4 Hz, 2H), 1.20 (t, 4 Hz, 2H).

^{13}C NMR (125 MHz, $CDCl_3$) δ 171.76, 82.55, 68.94, 60.68, 33.37, 14.34, 14.18. ESI MS: [M+H]+ calculated: 126.16, [M+H]- found: 123.3.

(viii) 3-(pent-4-ynoyloxy)-4-(trimethylammonio) butanoate (11)

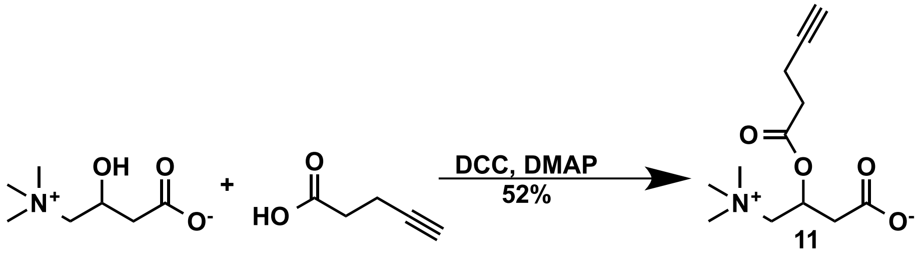

4-pentynoic acid (98 mg, 1 mmol), carnitine (HCl) (210 mg, 1.1 mmol) and DMAP (5 mg, 0.04 mmol) were dissolved in anhydrous DMF and chilled using an ice bath. DCC (229 mg, 1.1 mmol) was added and stirred for 10 minutes. The ice bath was removed and the reaction was stirred overnight at room temperature. The reaction mixture was filtered and DMF was removed *in vacuo*. The purified compound was isolated by HPLC using a mobile phase consisting of a gradient of MeCN in 0.1% trifluoroacetic acid (aqueous). HPLC purification was performed using an Agilent 1250 Infinity HPLC equipped with a semi-preparative Phenomenex Gemini C18 column (150 x 21.2 mm, 10 µm) to yield product **11** as an oil (125 mg, 52%). ^{1}H NMR (400 MHz, D_2O) δ 5.65 (q, 8 Hz, 1H), 3.87 (dd, 4 Hz, 4 Hz, 1H), 3.63 (d, 12 Hz, 1H), 3.13 (s, 9H), 2.80 (m, 2H), 2.62 (t, 8 Hz, 2H), 2.46 (t, 8 Hz, 2H), 2.30 (t, 4 Hz, 1H). ^{13}C NMR (101 MHz, D_2O) δ 173.13, 172.89, 83.29, 70.10, 67.68, 65.61, 53.83, 36.84, 33.04, 13.51. ESI MS: [M+H]+ calculated: 241.29, [M+H]+ found: 242.3.

(ix) propane-1,2,3-triyl tris(pent-4-ynoate) (12)

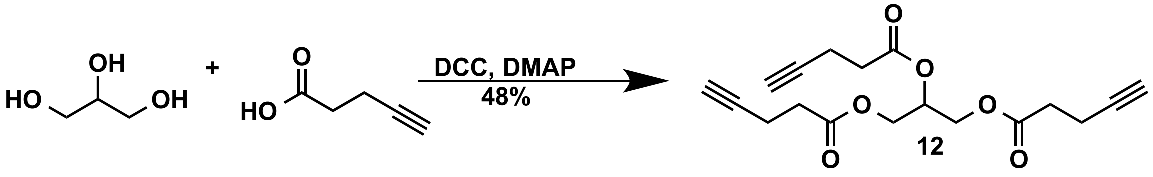

4-pentynoic acid (147 mg, 1.5 mmol), glycerol (46 mg, 0.5 mmol) and DMAP (5 mg, 0.04 mmol) were dissolved in anhydrous DMF and chilled using an ice bath. DCC (345 mg, 1.65 mmol) was added and stirred for 10 minutes. The ice bath was removed and the reaction was stirred overnight at room temperature. The reaction mixture was filtered and DMF was removed in vacuo. (0→30% CH_2Cl_2: MeOH) to yield product **12** as an oil (159 mg, 48%). ^{1}H NMR (400 MHz, $CDCl_3$) δ 5.29 (m, 1H), 4.45 (dd, J = 8 Hz, 4 Hz, 2H), 4.26 (dd, J = 4 Hz, 4H), 2.63 (m, 6H), 2.54 (m, 6H), 2.02 (t, J = 4 Hz, 3H). ^{13}C NMR (101 MHz, $CDCl_3$) δ 171.16, 170.77, 82.16, 82.02, 69.31, 69.26, 62.53, 33.22, 33.14, 14.35, 14.33.
ESI MS: [M+H]+ calculated: 332.35, [M+H]- found: 331.4.

(x) ethyl hex-5-ynoate (14)

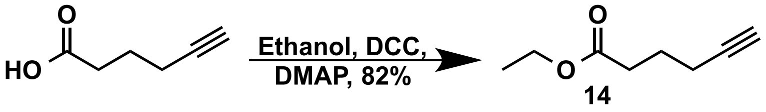

5-hexynoic acid (110 μL, 1 mmol), ethanol (117 μL, 2 mmol) and DMAP (5 mg, 0.04 mmol) were dissolved in anhydrous DMF and chilled using an ice bath. DCC (229 mg, 1.1 mmol) was added and stirred for 10 minutes. The ice bath was removed and the reaction was stirred overnight at room temperature. The reaction mixture was filtered and DMF was removed *in vacuo*. The purified

compound was isolated by flash chromatography (0→100% hexane: ethyl acetate) to yield product **14** as an oil (115 mg, 82%). ^{1}H NMR (400 MHz, $CDCl_3$) δ 4.49 (q, *J* = 8 Hz 2H), 2.82 (t, *J* = 8Hz, 2H), 2.38 (t, *J* = 4 Hz, 2H), 2.03, (m, 3H), 1.44 (t, *J* = 8Hz, 3H). ^{13}C NMR (101 MHz, $CDCl_3$) δ 173.09, 83.30, 69.05, 60.38, 32.95, 23.64, 17.85, 14.21. ESI MS: [M+H]+ calculated: 140.18, [M+H]- found: 137.1.

(xI) 3-(hex-5-ynoyloxy)-4-(trimethylammonio)butanoate (15)

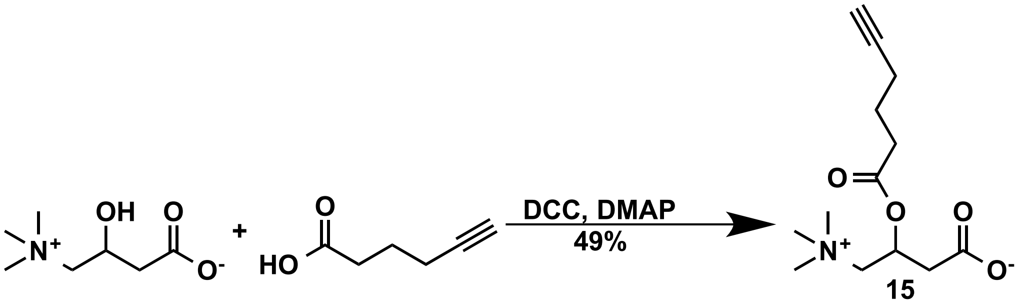

5-hexynoic acid (110 μL, 1 mmol), carnitine (HCl) (322 mg, 2 mmol) and DMAP (3 mg, 0.02 mmol) were dissolved in anhydrous DMF and chilled using an ice bath. DCC (229 mg, 1.1 mmol) was added and stirred for 10 minutes. The ice bath was removed and the reaction was stirred overnight at room temperature. The reaction mixture was filtered and DMF was removed in vacuo. The purified compound was isolated by HPLC using a mobile phase consisting of a gradient of MeCN in 0.1% trifluoroacetic acid (aqueous). HPLC purification was performed using an Agilent 1250 Infinity HPLC equipped with a semi-preparative Phenomenex Gemini C18 column (150 x 21.2 mm, 10 μm) to yield product **15** as an oil (112 mg, 44%). ^{1}H NMR (400 MHz, D_2O) δ 5.60 (q, *J* = 4 Hz, 1H), 3.82 (dd, *J* = 8 Hz, 4Hz 1H), 3.61, (d, *J* = 16 Hz, 1H), 3.12 (s, 9H), 2.77 (m, 2H), 2.51 (t, *J*

= 8 Hz, 2H), 2.29 (t, *J* = 2Hz, 1H), 2.19 (td, *J* = 4Hz, 2Hz, 2H), 1.71 (q, *J* = 8Hz, 2H). ^{13}C NMR (101 MHz, D_2O) δ 174.35, 172.89, 84.45, 70.03, 67.66, 65.34, 53.81, 36.81, 32.64, 22.58, 16.93. ESI MS: [M+H]+ calculated: 255.31, [M+H]+ found: 256.2.

(xii) propane-1,2,3-triyl tris(hex-5-ynoate) (16)

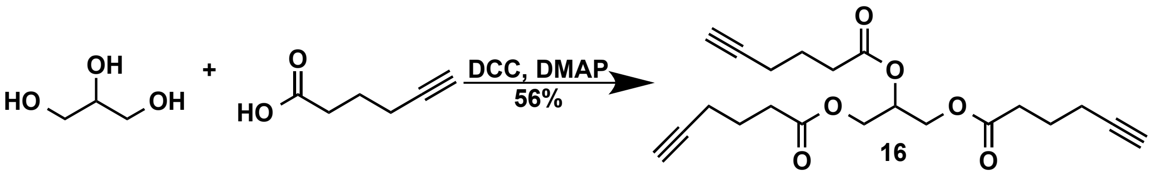

5-hexynoic acid (330 μL, 3 mmol), glycerol (46 mg, 0.5 mmol) and DMAP (5 mg, 0.04 mmol) were dissolved in anhydrous DMF and chilled using an ice bath. DCC (345 mg, 1.65 mmol) was added and stirred for 10 minutes. The ice bath was removed and the reaction was stirred overnight at room temperature. The reaction mixture was filtered and DMF was removed in vacuo. (0→30% CH_2Cl_2: MeOH) to yield product **12** as an oil (159 mg, 85%). ^{1}H NMR (500 MHz, $CDCl_3$) δ 5.29 (m, 1H), 4.34 (dd, *J* = 10 Hz, 5 Hz, 2H), 4.17 (dd, *J* = 10 Hz, 5 Hz, 2H), 2.50 (m, 6H), 2.28 (m, 6H), 2.00 (t, *J* = 5 Hz, 3H), 1.87 (m, 6H). ^{13}C NMR (125 MHz, $CDCl_3$) δ 172.52, 172.14, 83.06, 83.05, 77.29, 77.24, 77.04, 76.79, 69.32, 69.30, 69.00, 62.19, 32.76, 32.57, 23.49, 23.44, 17.77, 17.75. ESI MS: [M+H]+ calculated: 374.43, [M+H]+ found: 375.4.

^{1}H- and ^{13}C-NMR spectra for pro-metabolites

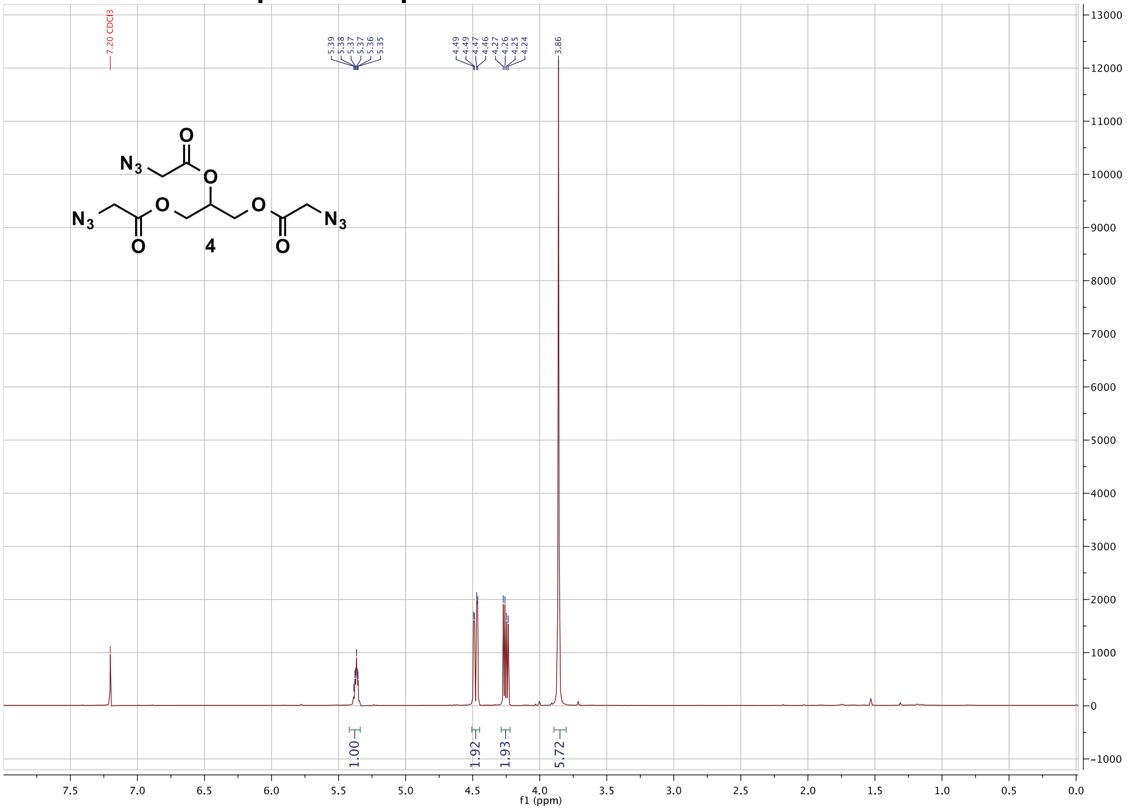

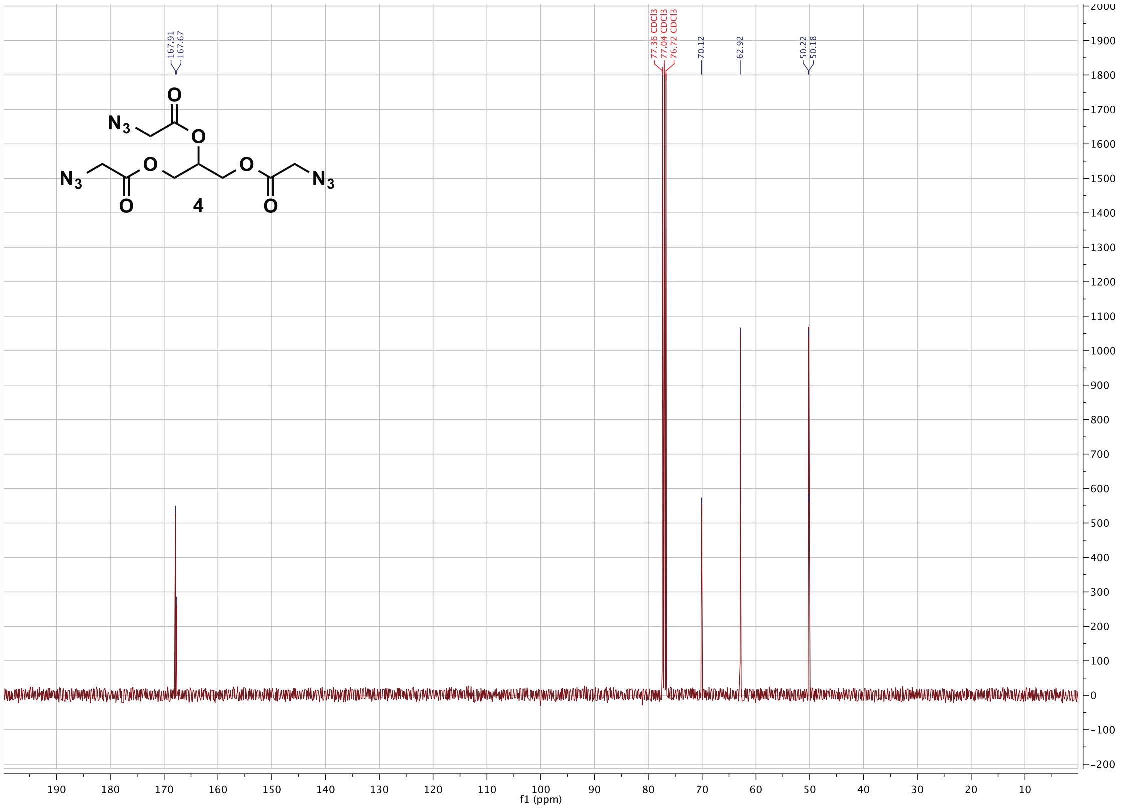

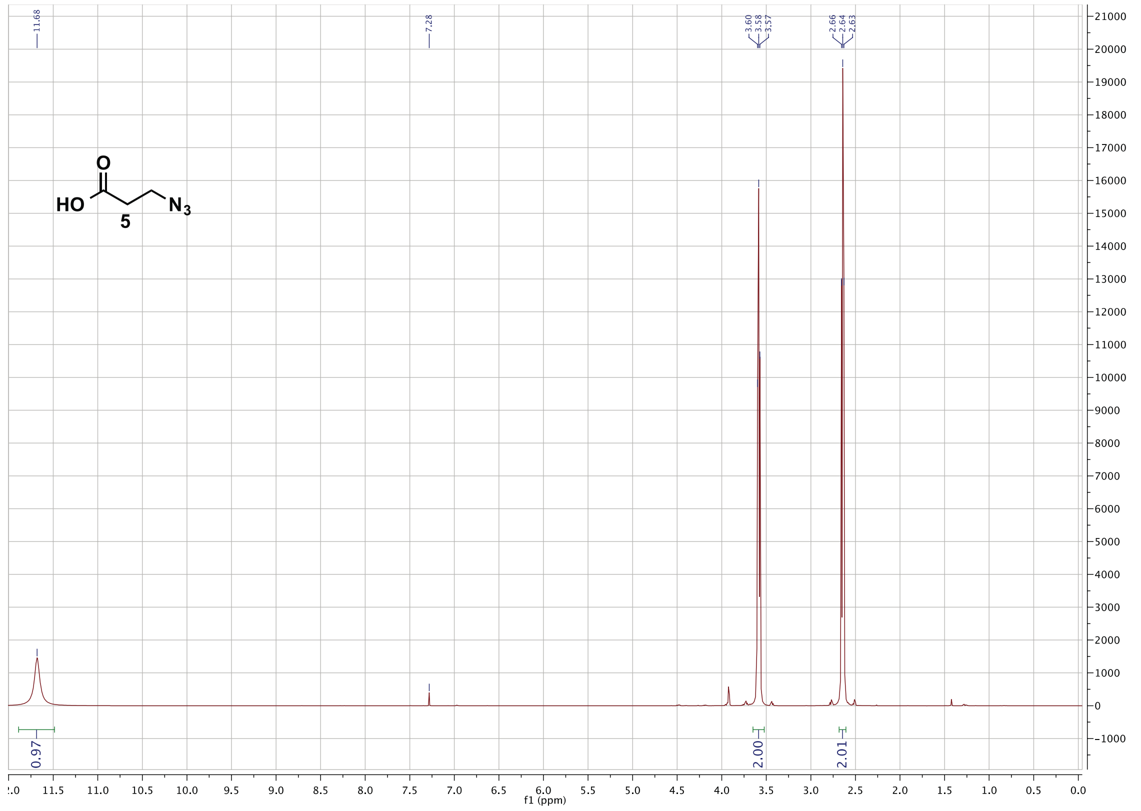

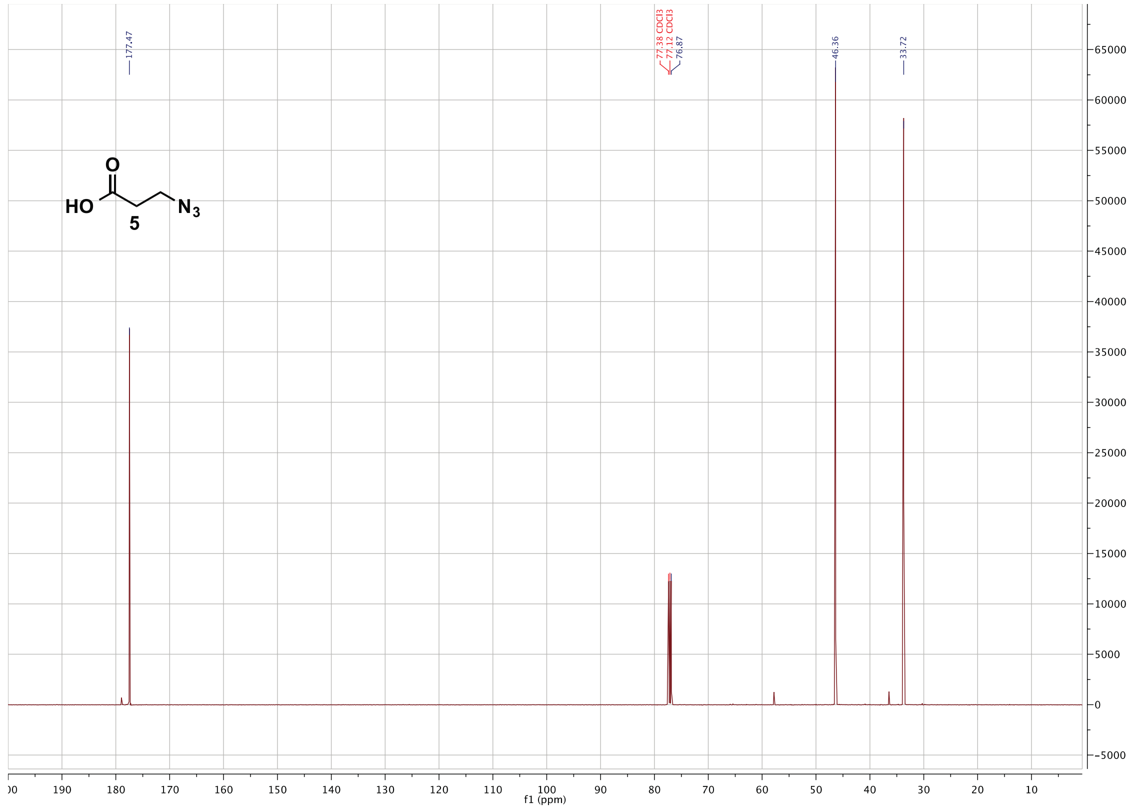

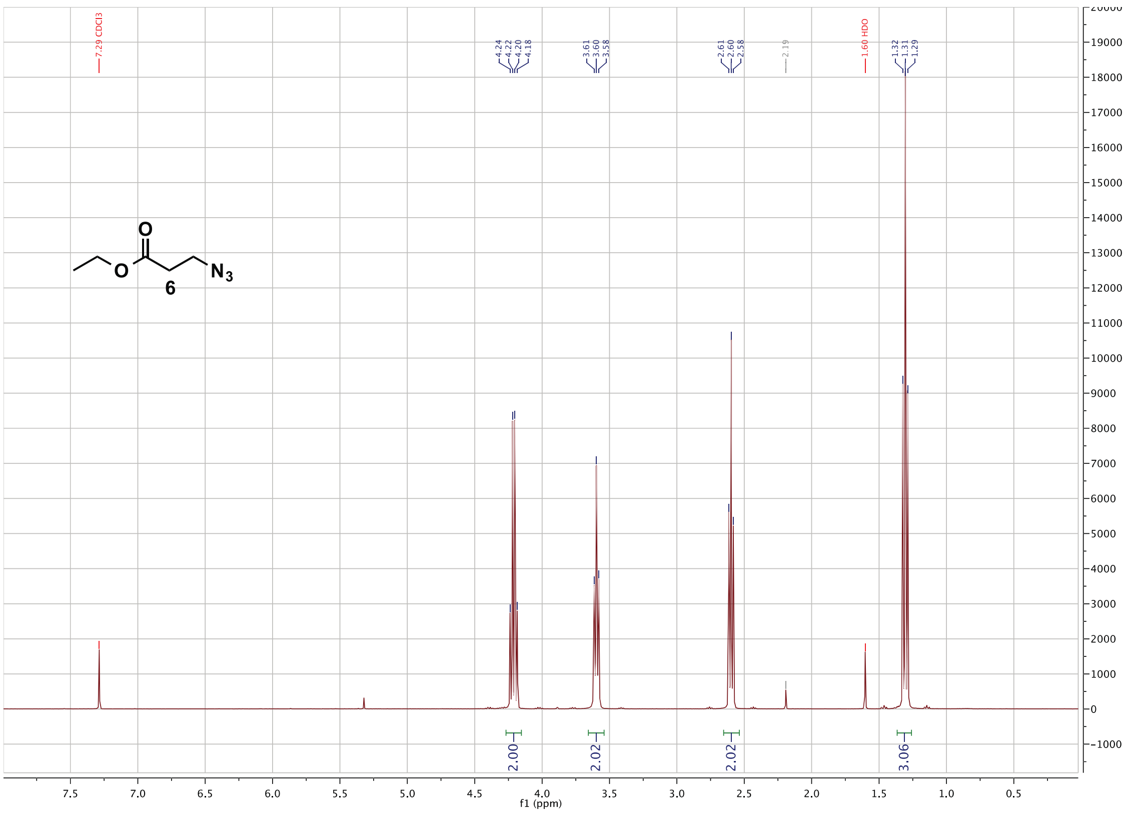
7.29 CDCl3
4.24
4.22
4.20
4.18
3.61
3.60
3.58
2.61
2.60
2.58
2.19
1.60 HDO
1.32
1.31
1.29
O
O
N3
6
2.00
2.02
2.02
3.06
f1 (ppm)

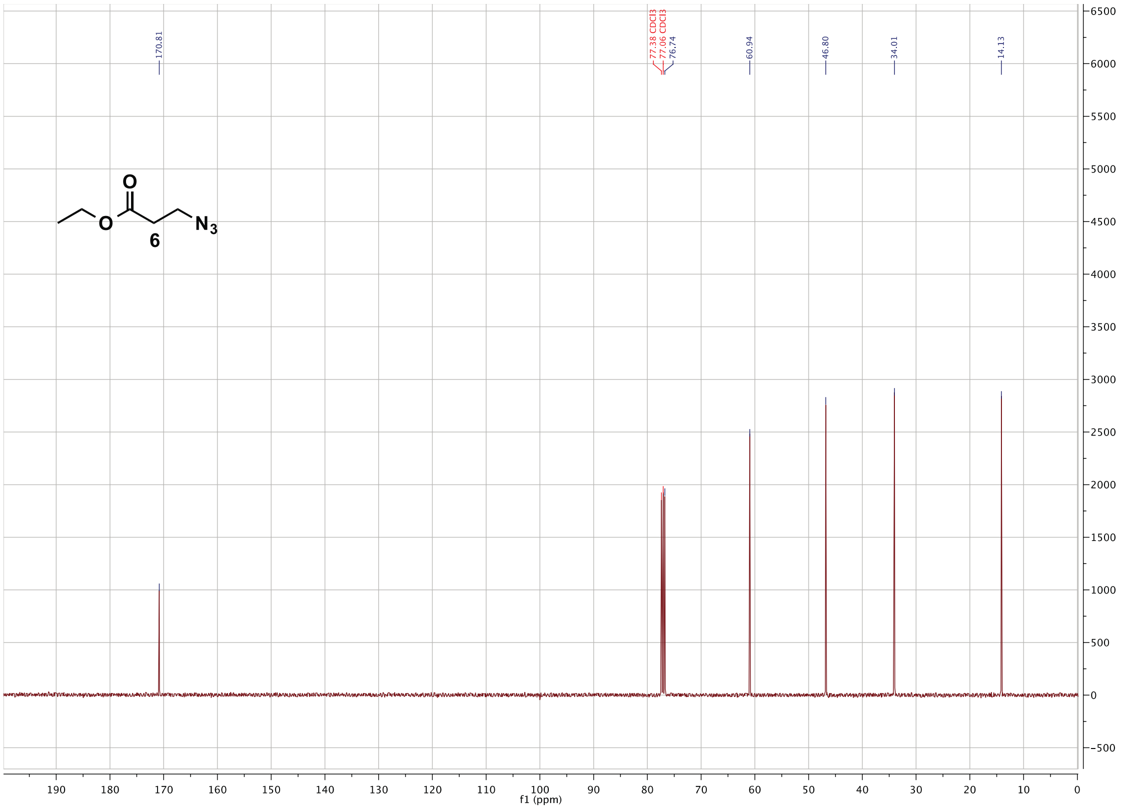
170.81
77.38 CDCl3
77.06 CDCl3
76.74
60.94
46.80
34.01
14.13
O
O
N3
6
f1 (ppm)

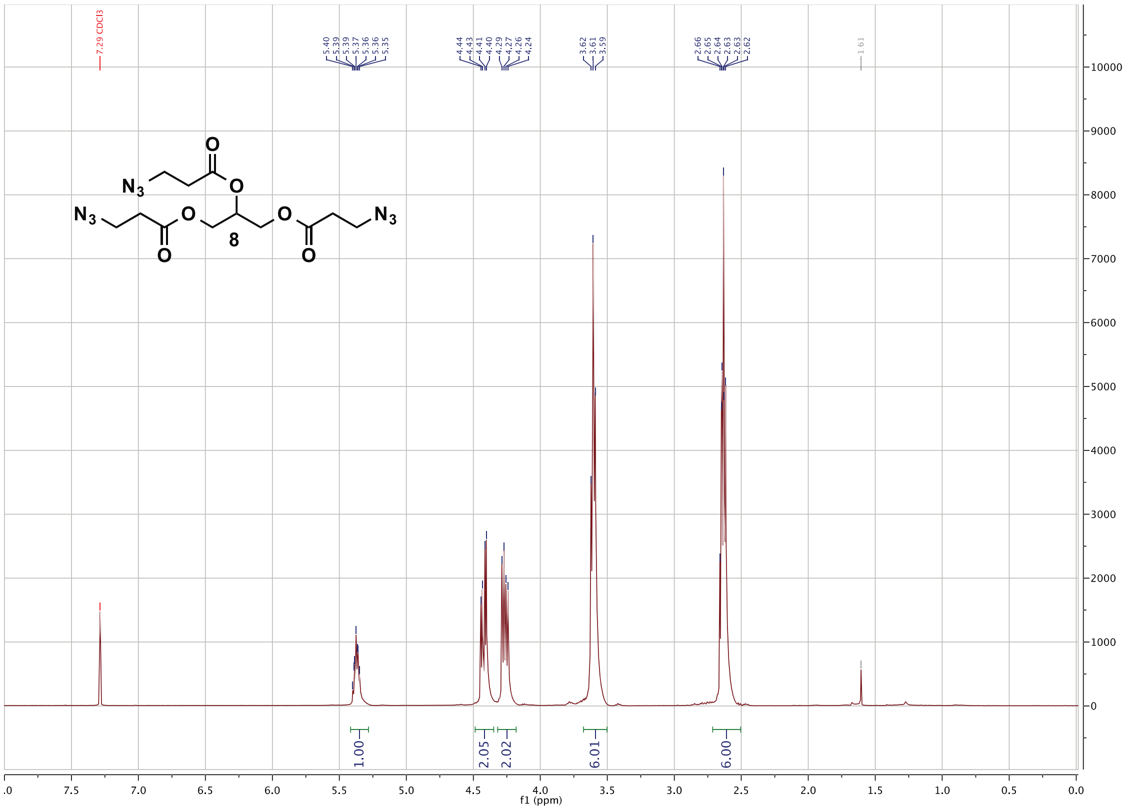

7.29 CDCl3
5.40
5.39
5.39
5.37
5.36
5.36
5.35
4.44
4.43
4.41
4.40
4.29
4.27
4.26
4.24
3.62
3.61
3.59
2.66
2.65
2.64
2.63
2.63
2.62
1.61
N3
O
O
N3
O
O
O
O
N3
8
1.00
2.05
2.02
6.01
6.00
f1 (ppm)

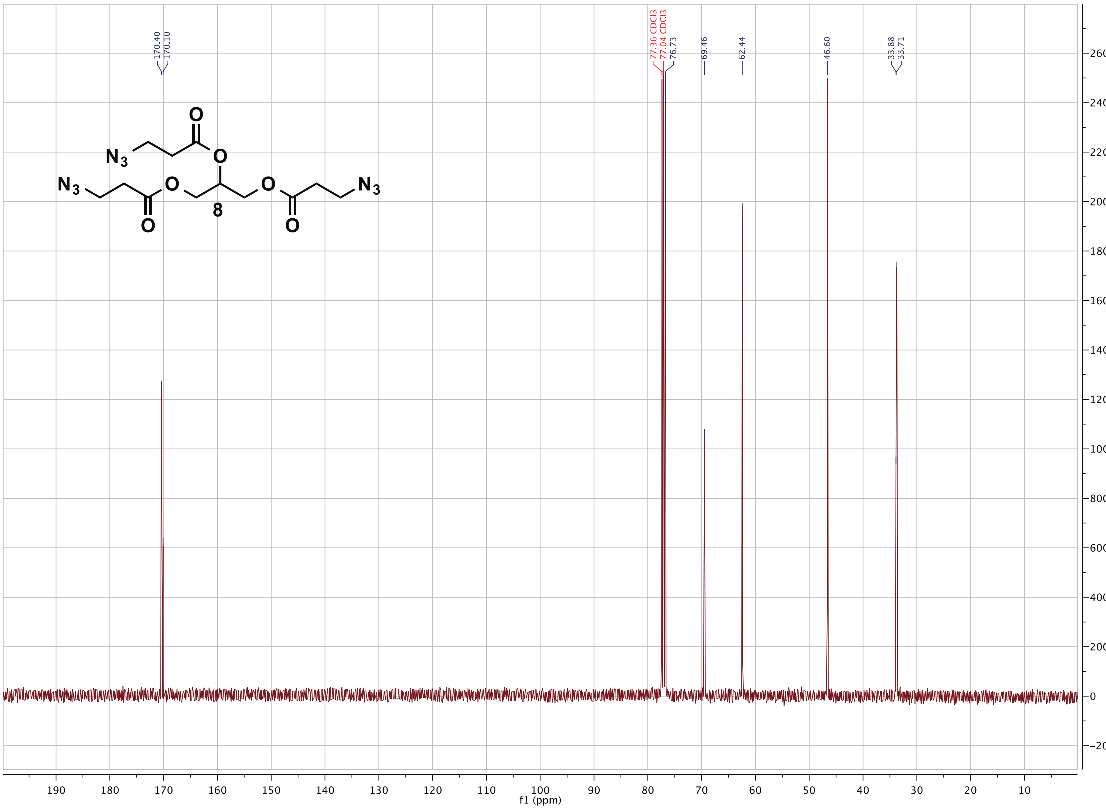

170.40
170.10
77.36 CDCl3
77.04 CDCl3
76.73
69.46
62.44
46.60
33.88
33.71
N3
O
O
N3
O
O
O
O
N3
8
f1 (ppm)

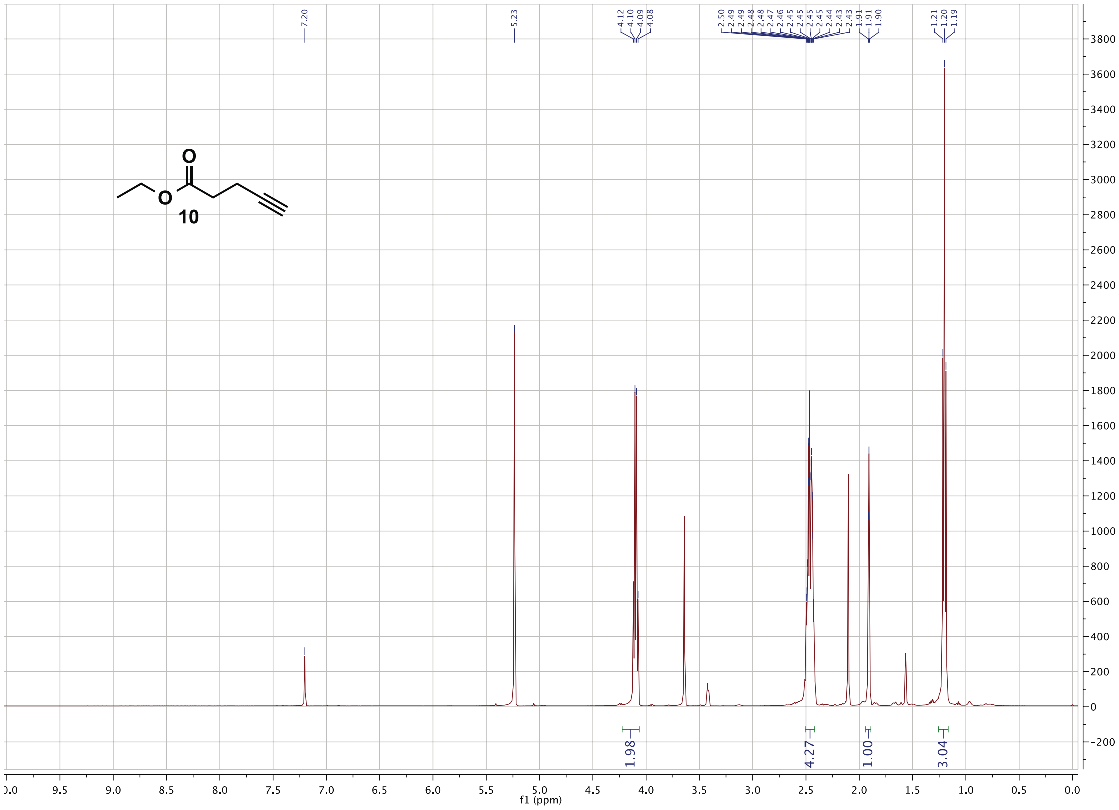

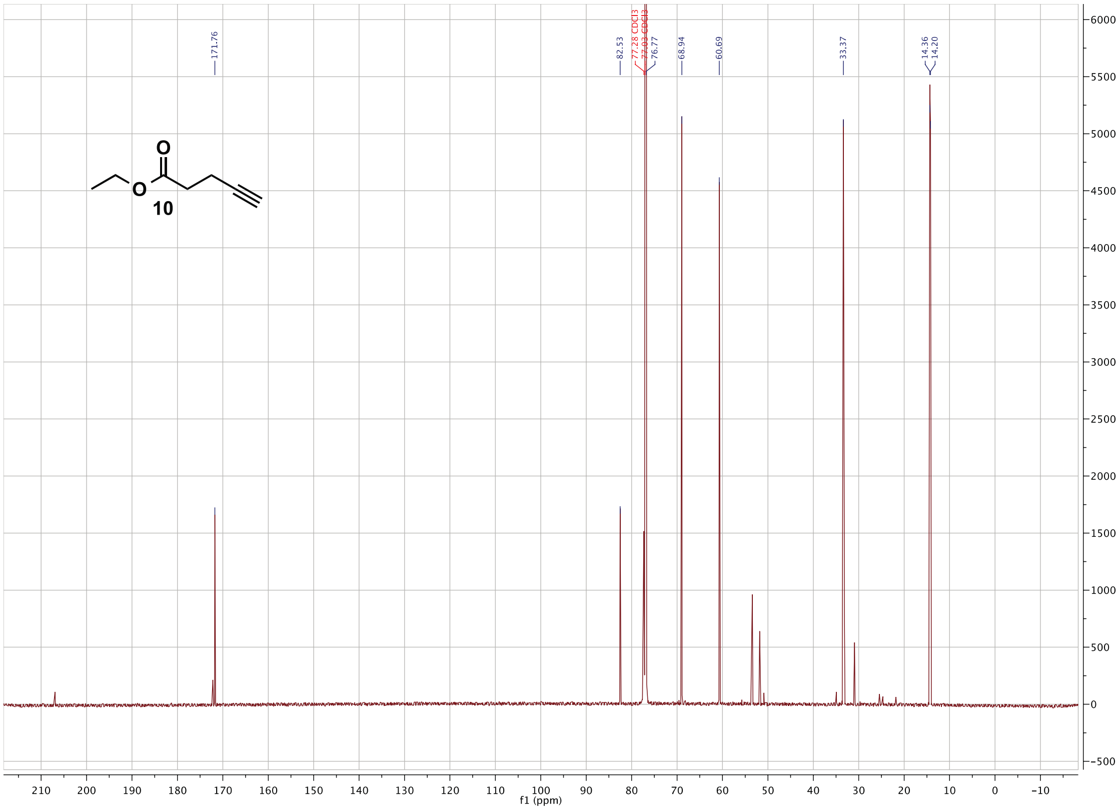

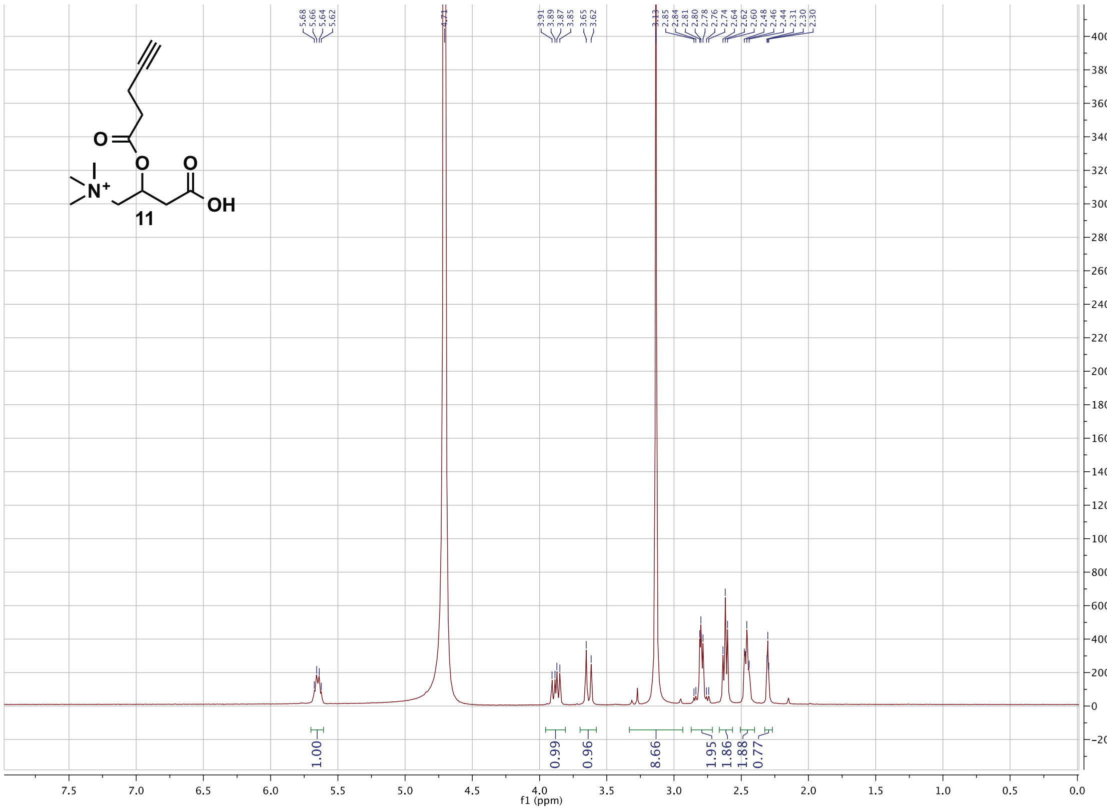
5.68
5.66
5.64
5.62
4.71
3.91
3.89
3.87
3.85
3.65
3.62
3.13
2.85
2.84
2.81
2.80
2.78
2.76
2.74
2.64
2.62
2.60
2.48
2.46
2.44
2.31
2.30
2.30
1.00
0.99
0.96
8.66
1.95
1.86
1.88
0.77
11
OH
f1 (ppm)

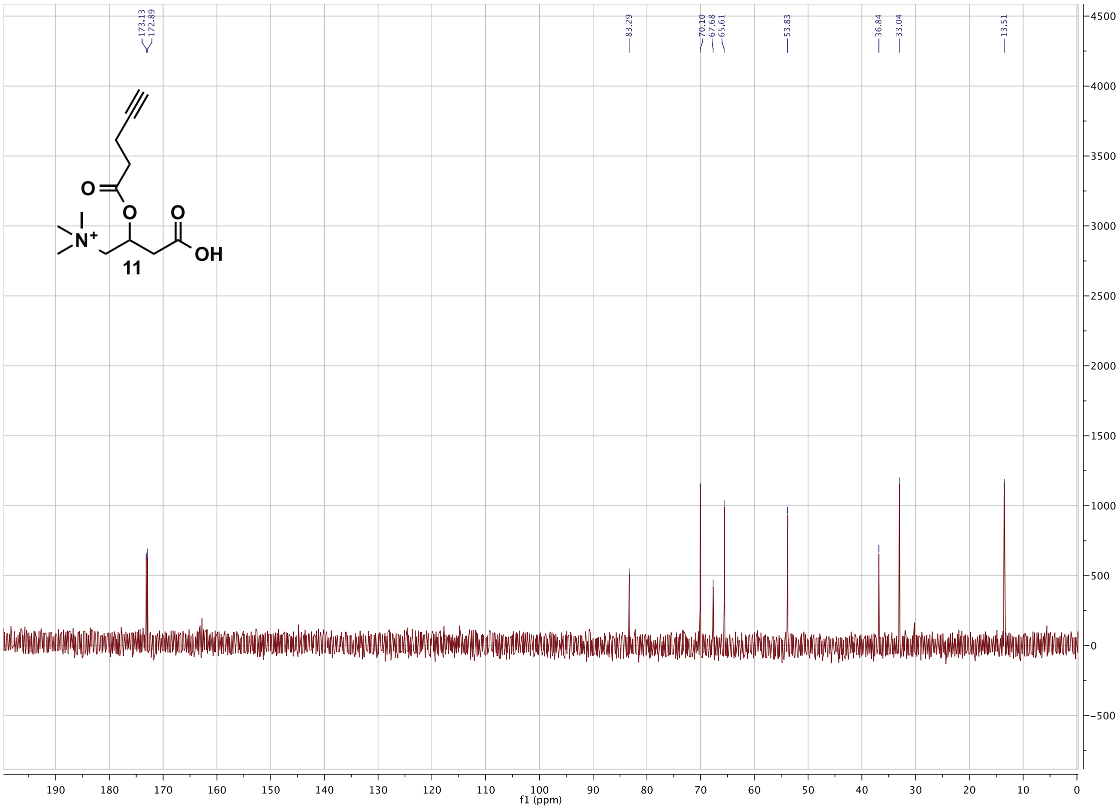
173.13
172.89
83.29
70.10
67.68
65.61
53.83
36.84
33.04
13.51
11
OH
f1 (ppm)

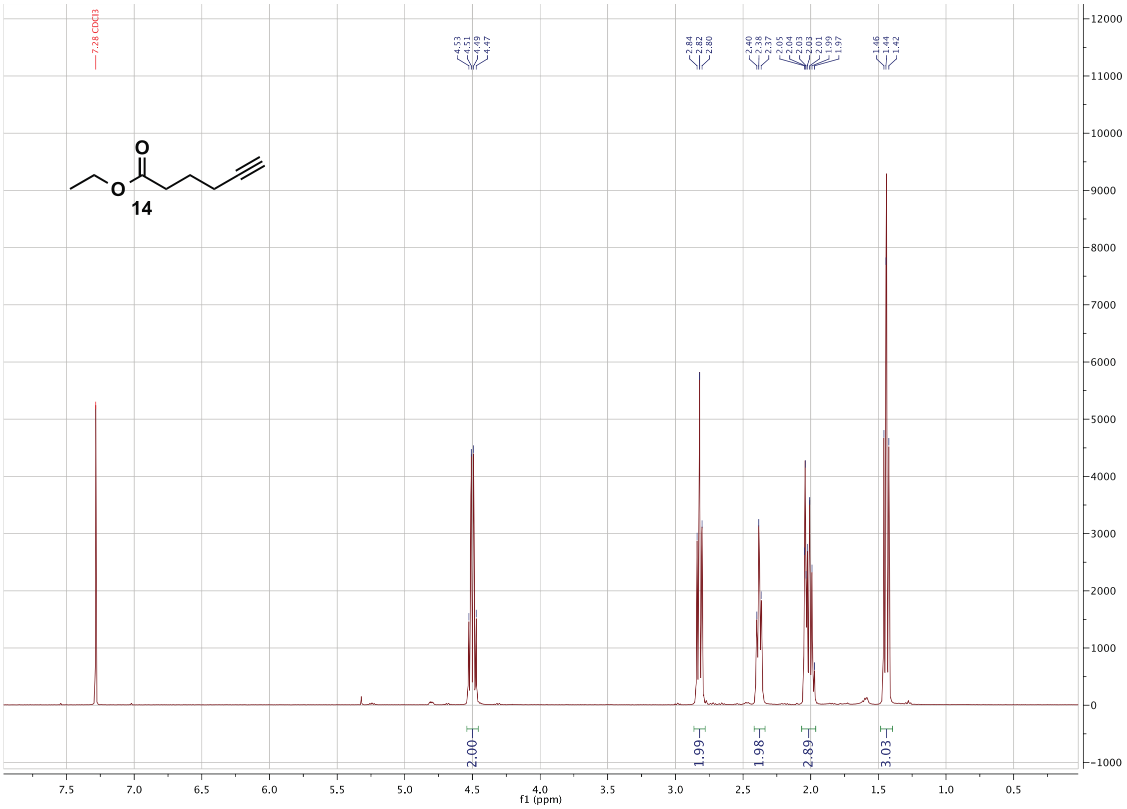
7.28 CDCl3
4.53
4.51
4.49
4.47
2.84
2.82
2.80
2.40
2.38
2.37
2.05
2.04
2.03
2.03
2.01
1.99
1.97
1.46
1.44
1.42
O
O
14
2.00
1.99
1.98
2.89
3.03
f1 (ppm)

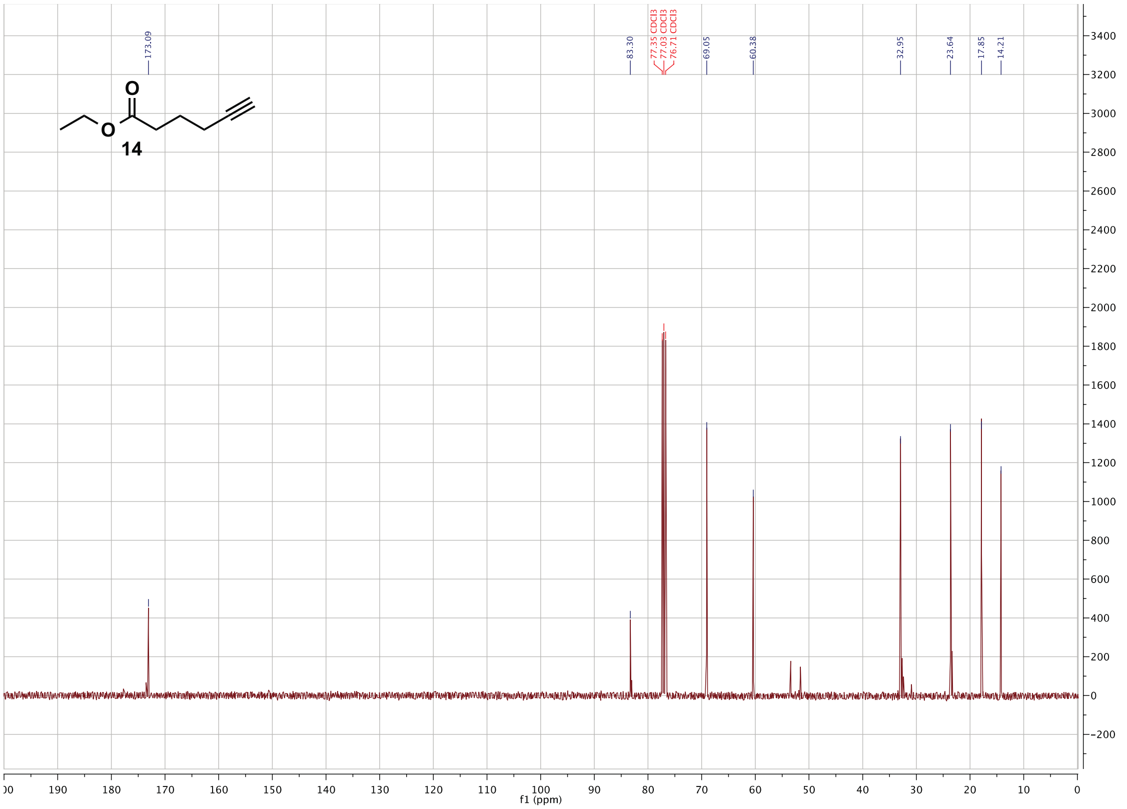
173.09
83.30
77.35 CDCl3
77.03 CDCl3
76.71 CDCl3
69.05
60.38
32.95
23.64
17.85
14.21
O
O
14
f1 (ppm)

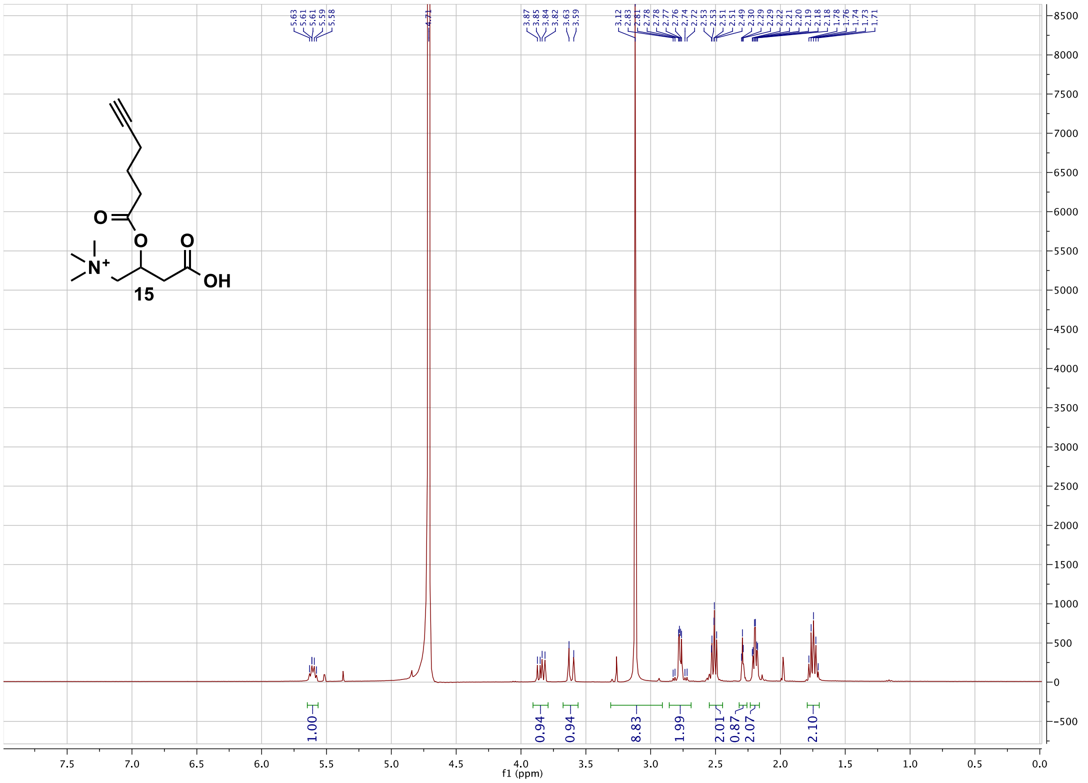

O
O
O
OH
N+
15
1.00
0.94
0.94
8.83
1.99
2.01
0.87
2.07
2.10
f1 (ppm)

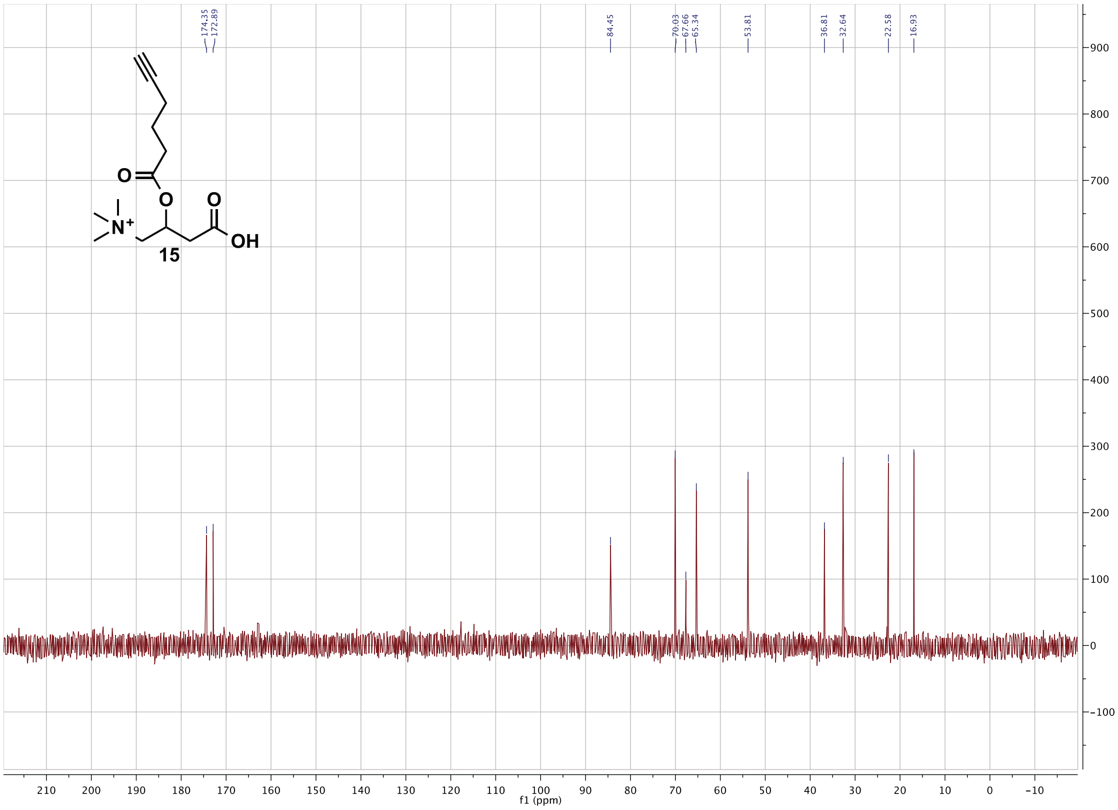

174.35
172.89
84.45
70.03
67.66
65.34
53.81
36.81
32.64
22.58
16.93
O
O
O
OH
N+
15
f1 (ppm)

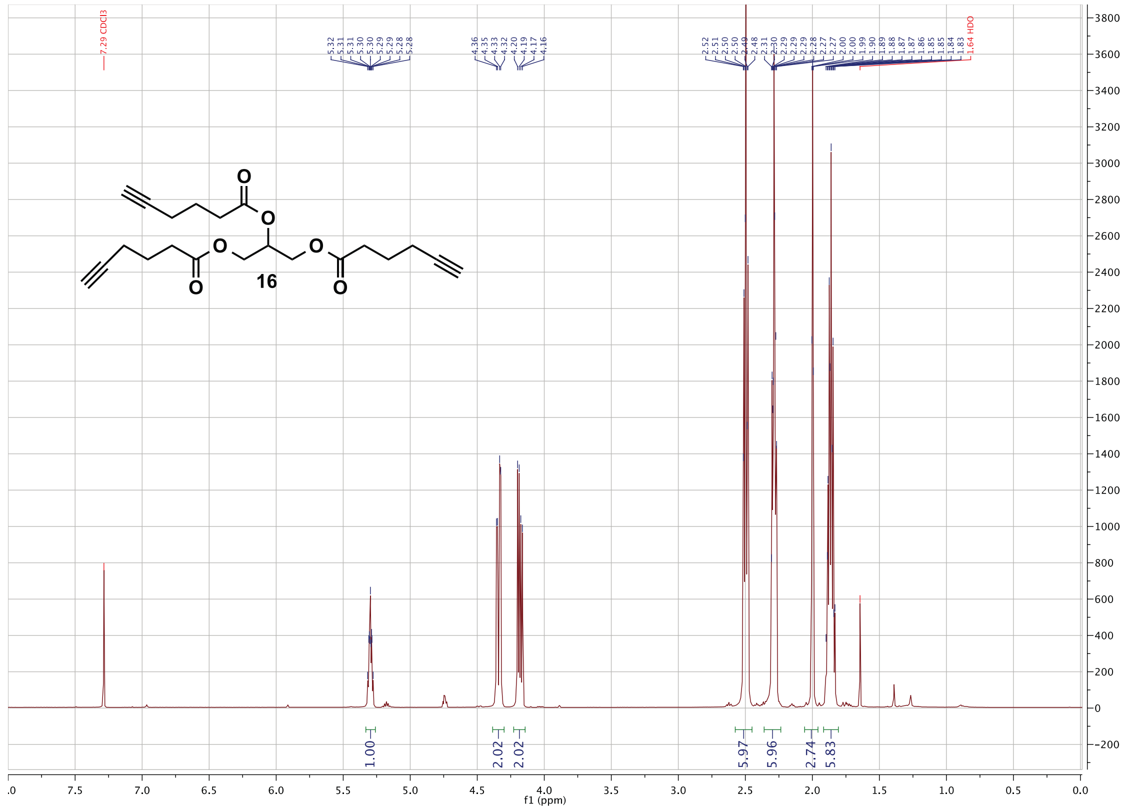

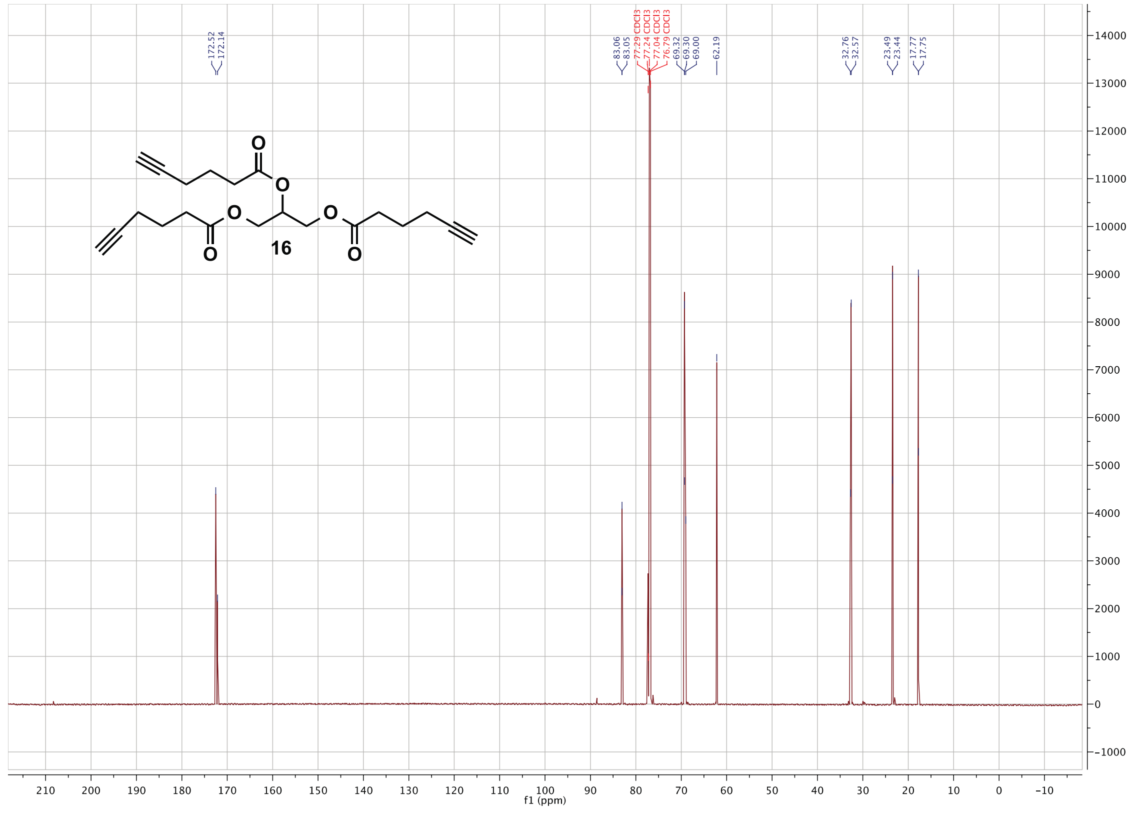

General procedures and materials for cellular assays

A549 and HEK293T cells were obtained from the NCI Tumor Cell Repository, while HepG2 cells were obtained from ATCC (Manassas VA). All cell lines were cultured at 37 °C under 5% CO_2 atmosphere in a growth medium of RPMI supplemented with 10% FBS and 2 mM glutamine, with the exception of HEK293T cell lines, which were cultured in DMEM supplemented with 10% FBS, 2 mM glutamine. Cells were harvested by scraping and cell lysates prepared by sonication as previously described.[2] Protein concentrations were determined by Qubit Protein Assay kit (Life Technologies #Q33212). Fluorescent labeling analyses were performed via Cu-catalyzed ligation to a TAMRA-azide or alkyne as previously described.[3] SDS-PAGE was performed using Bis-Tris NuPAGE gels (4-12%, Invitrogen #NP0322), and MES running buffer (Life technologies #NP0002) in Xcell SureLock MiniCells (Invitrogen) according to the manufacturer's instructions. SDS-PAGE fluorescence was visualized using an ImageQuant Las4010 Digitial Imaging System (GE Healthcare). Total protein content on SDS-PAGE gels was visualized by Blue-silver Coomassie stain.[4] SILEC experiments were performed as previously described. Acyl-CoAs were isolated by acid extraction and analyzed by LC-MS as previously described.[5, 6] 3-azidopropionyl-CoA was synthesized and purified by standard methods,[2, 7] and used as a standard in LC-MS analyses. Histones were isolated by acid extraction and analyzed by immunoblotting as previously described.[8, 9] Toxicity of pro-metabolites were assessed by sulforhodamine B staining[10] after treatment for the dose/duration indicated. For proteomic analyses, HEK293 proteomes (8 mg per

replicate, treated with **6** or vehicle DMSO) were ligated to a biotin-alkyne using Cu-catalyzed [3+2] cycloaddition.[3, 11] Enrichment, tryptic digest, LC-MS/MS analyses, and database searching was performed as previously described.[3]

Treatment of cells with pro-metabolites for metabolic labeling analyses

For labeling experiments, cell lines were plated and allowed to adhere overnight. Cells were then treated with pro-metabolites **1-16** by adding of DMSO stock solutions directly to growth medium at the specified concentration, followed by gentle agitation and incubation for the specified time. For analysis of glucose levels on pro-metabolite labeling, cells were grown in either glucose-replete (4.8 g/L) or glucose-free DMEM for 24 h prior to addition of pro-metabolite **6**. For acetate experiments, acetate (5 mM) was added to media concurrently with pro-metabolite **6**. For p300i experiments, p300i (100 nM; Figure S5a) was added to media 24 h prior to addition of pro-metabolite **6.** For SAHA experiments, SAHA (10 μM) was added to media 2 h prior to addition of pro-metabolite **6**. In all experiments, final DMSO concentrations were < 0.5%. Unfractionated proteomes were harvested by washing adherent cells (80-90% confluent) 3x with ice cold PBS, and scraping cells into a Falcon tube followed by centrifugation (500 rcf x 5 min, 4 °C). Lysates were isolated from cell pellets by sonication as described above. Specific experimental treatment conditions are also indicated in the figure captions.

Mass spectrometry characterization of azidopropionyl-CoA formation

Cellular formation of azidopropionyl-CoA was confirmed by LC-HRMS and LC-MS/HRMS using previously described methods.[12] Briefly, azidopropionyl-CoA ($C_{24}H_{39}N_{10}O_{17}P_3S$), generated synthetically or by cell culture treatment in HepG2 cells using **5** or **6** was observed as an $[M+H]^+$ (predicted *m/z* 865.1501, observed in cell culture extract 865.1505 dppm = 0.46) (Fig. S3b-c). Predominant fragmentation in MS/HRMS was derived from the neutral loss of 507 mass units from the loss of the adenosine with the pantetheine ejection maintaining the charge ($[C_{14}H_{24}N_5O_4S]^+$ product predicted *m/z* 358.1544, observed 358.1543 dppm= -0.27) (Fig. S3a, Fig. S3c). To further confirm this finding, we performed stable isotope labeling by essential nutrients in cell culture (SILEC), treating Hepa1c1c7 murine hepatocellular carcinoma cells with $^{13}C_3$ $^{15}N_1$ pantothenate as previously reported.[13] Treatment of cells with **6** (0.1 mM, 1 h) generated $^{13}C_3$ $^{15}N_1$-azidopropionyl-CoA, where the stable isotope labeling is enriched in the region of the CoA backbone generated from pantothenate. A mixture of 1:1 by volume of cell extract from unlabeled and SILEC labeled cells produced a perfectly co-eluting HRMS and MS/HRMS peak, validating our identification of azidopropionyl-CoA as a biochemically produced acyl-CoA metabolite (Fig. S3d).

Uncropped gels, immunoblots, and loading controls

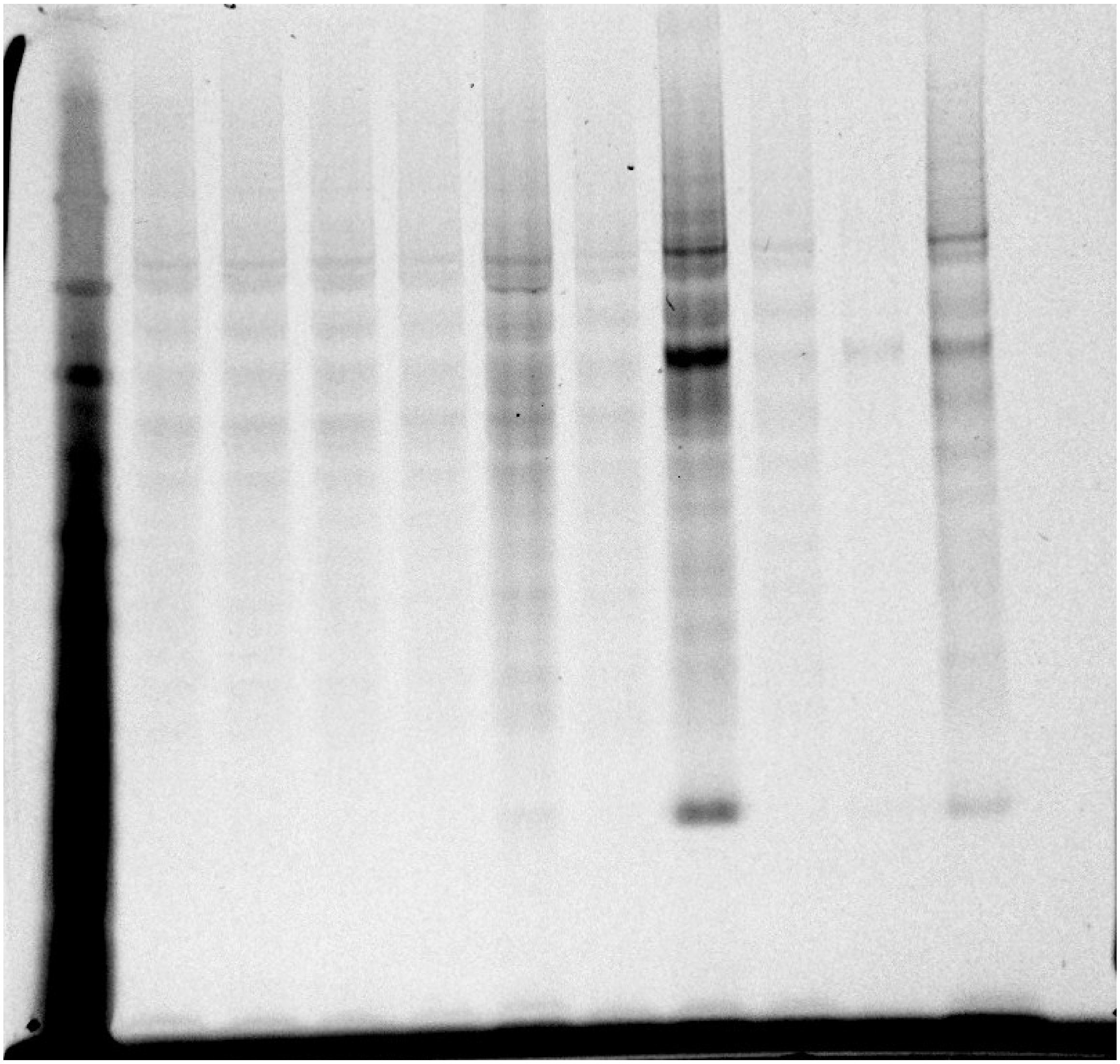

Figure 3b, compounds **1**-**8**, fluorescence

Lane 1 (ladder) and Lane 10 (2.5 mM **8**) were omitted from Figure 3. Lane 11 (1 mM **8**) was cropped and put next to lane 9.

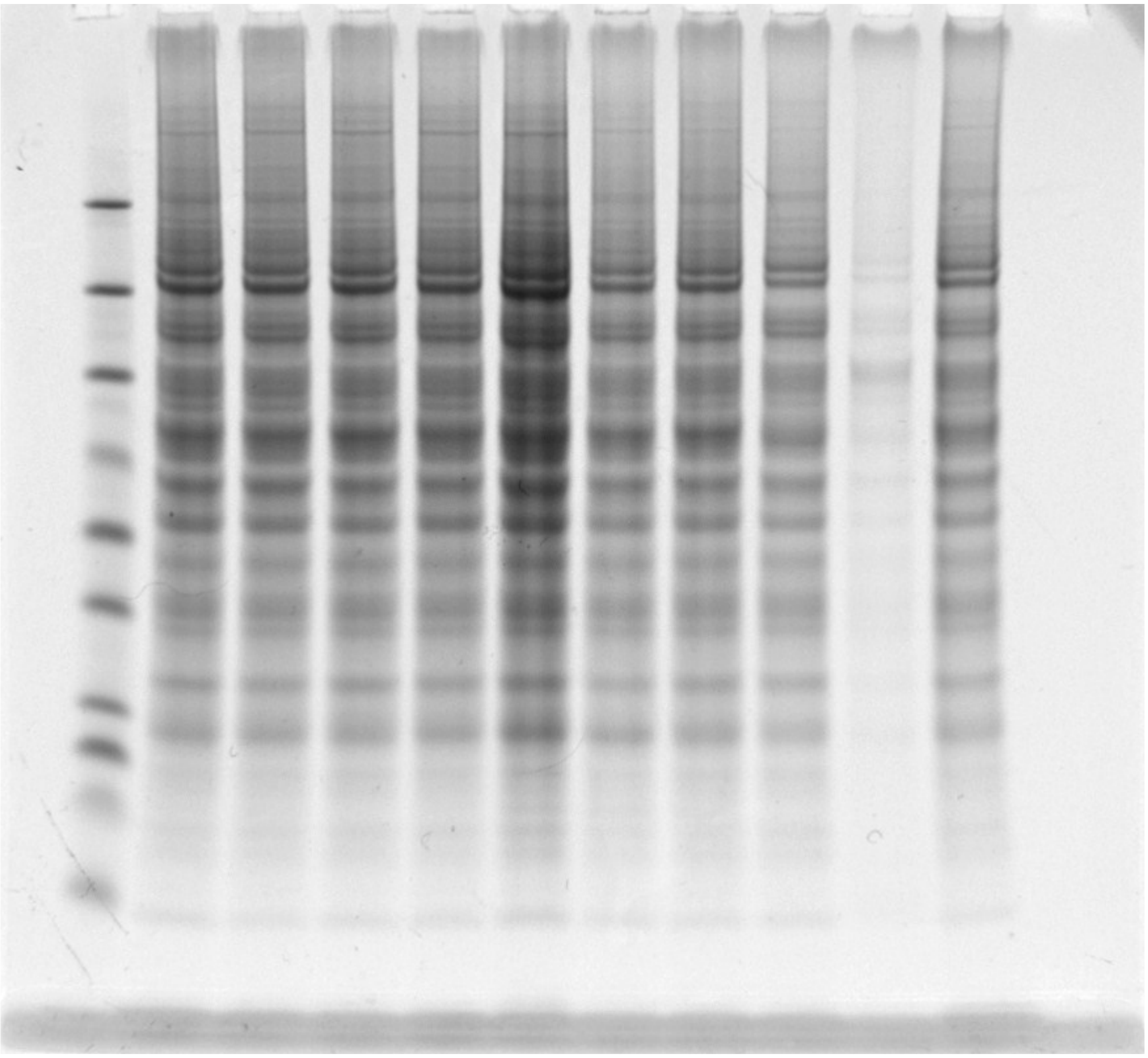

Figure 3b, compounds **1**-**8**, Coomassie

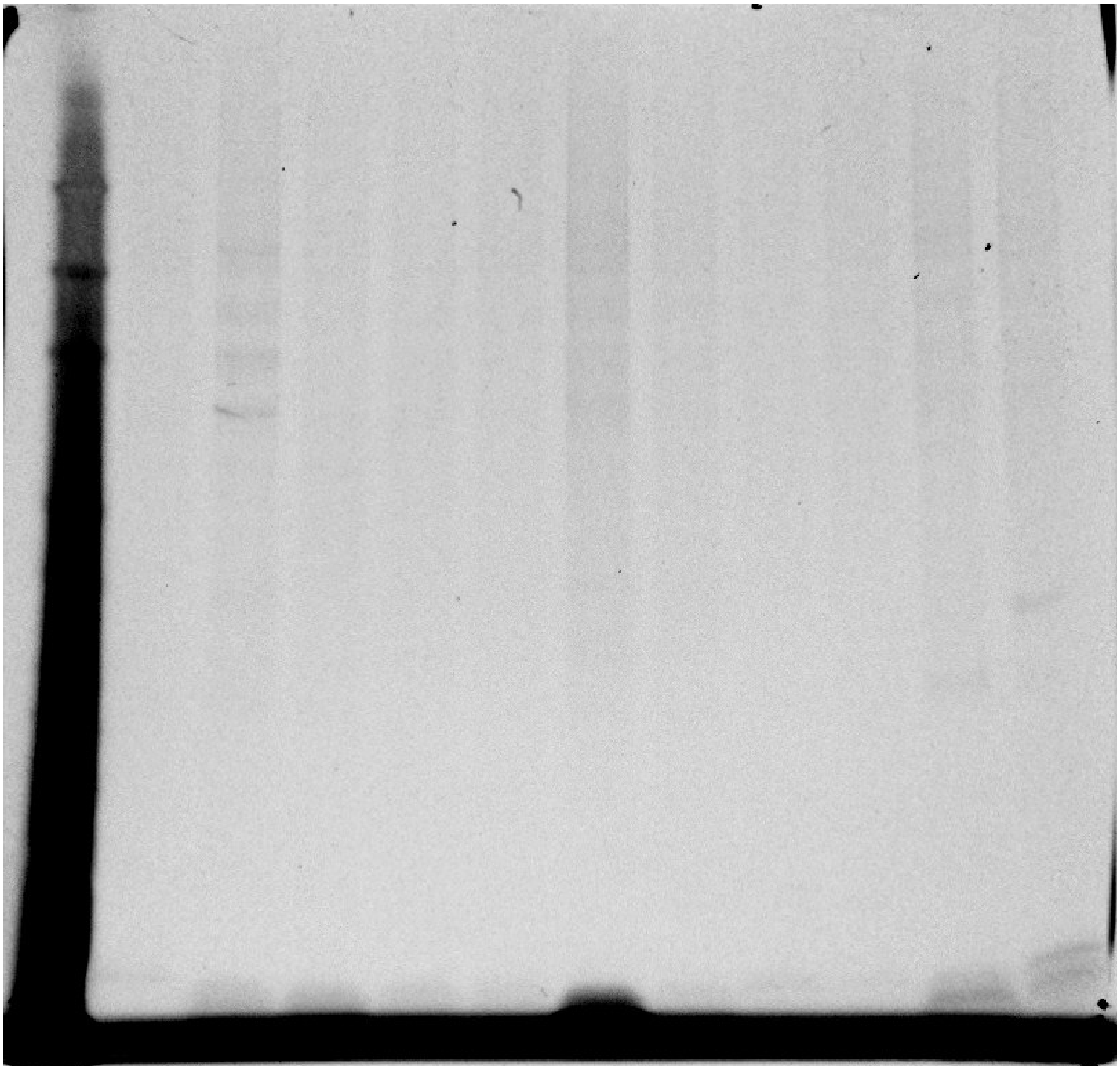

Figure 3b, compounds **9**-**16**, fluorescence

Lane 1 (ladder) and 3 (electrophilic alkyne used as positive control for labeling) were omitted from Figure 3. Lane 2 was cropped and put next to lane 4.

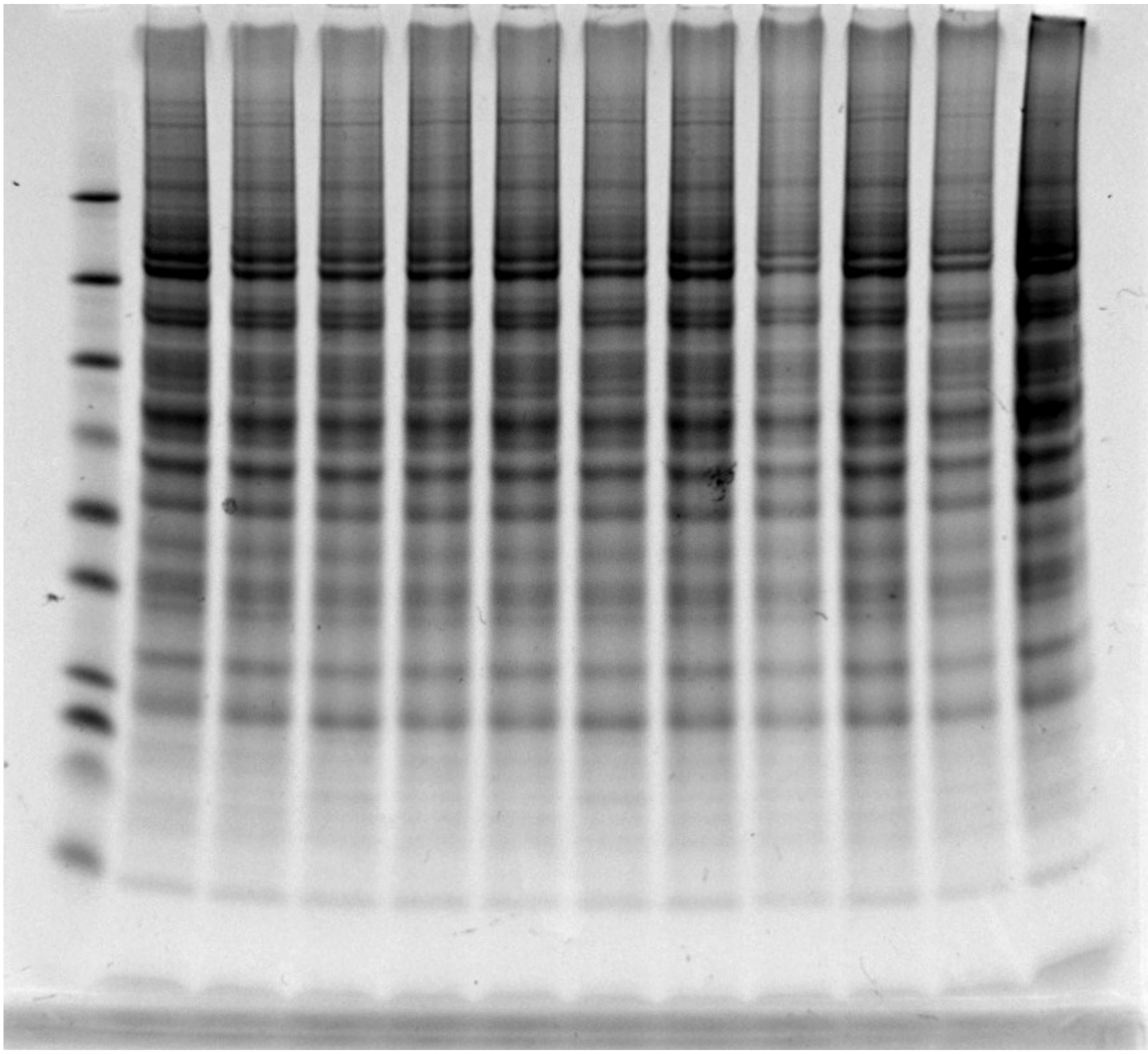

Figure 3b, compounds **9**-**16**, Coomassie

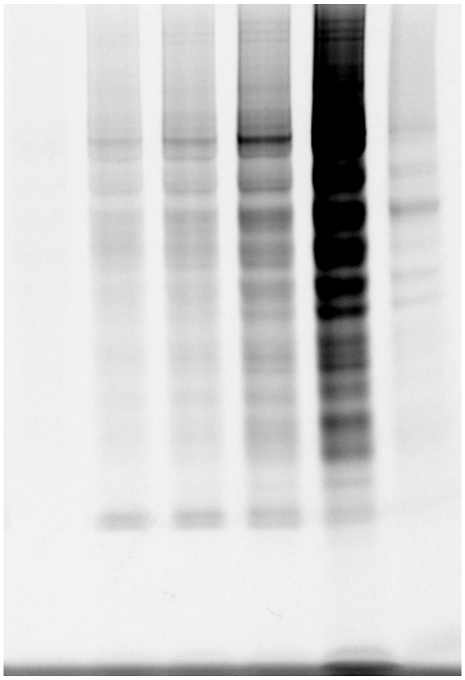

Figure 4a, dose-dependence, fluorescence

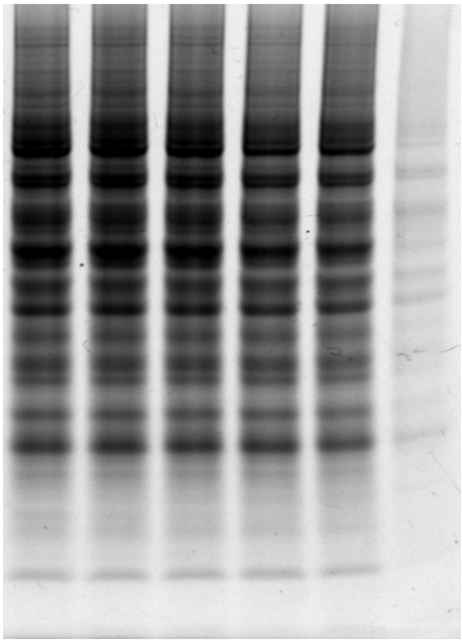

Figure 4a, dose-dependence, Coomassie

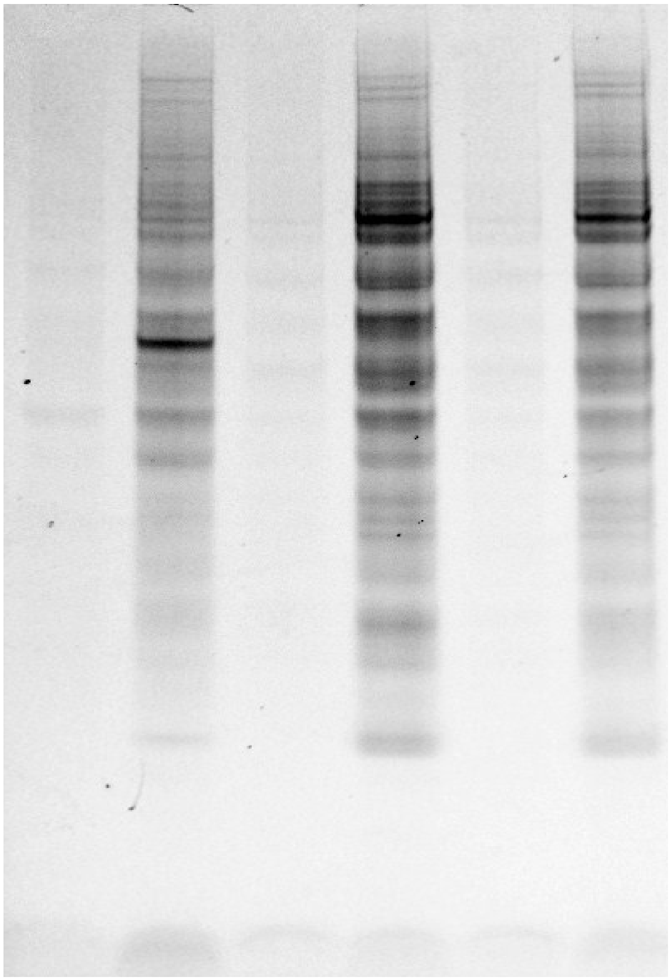

Figure 4b, cell lines, fluorescence

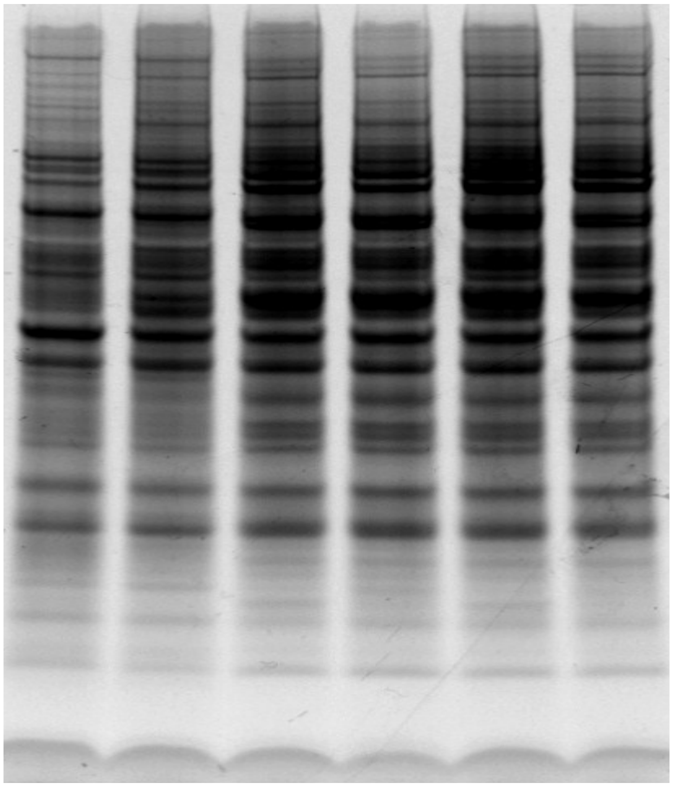

Figure 4b, cell lines, Coomassie

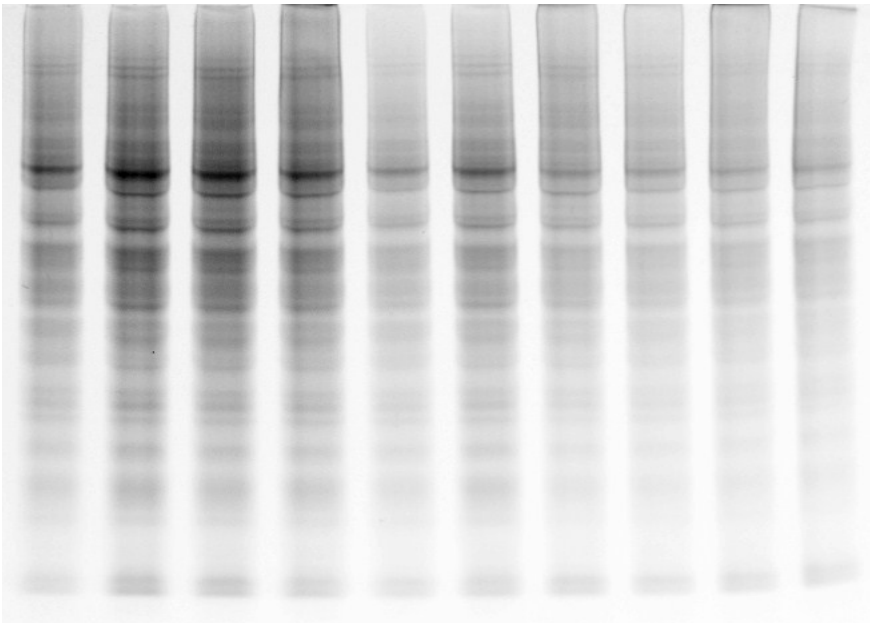

Figure 5b, acetate competition, fluorescence

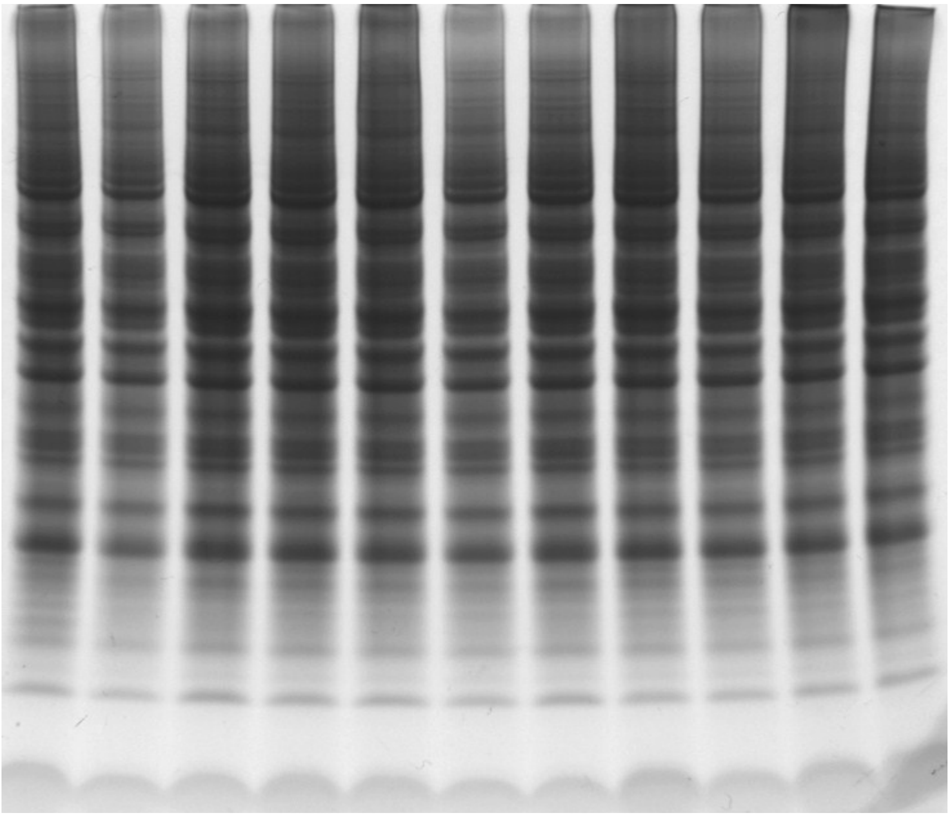

Figure 5b, acetate competition, Coomassie

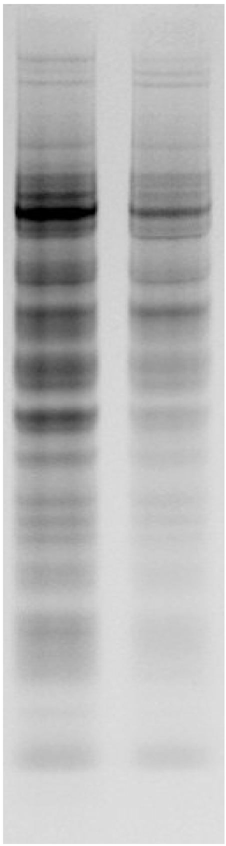

Figure 5c, glucose-dependence, HepG2, fluorescence

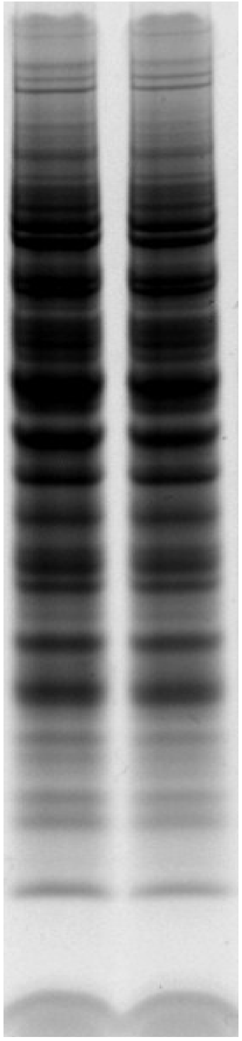

Figure 5c, glucose-dependence, HepG2, Coomassie

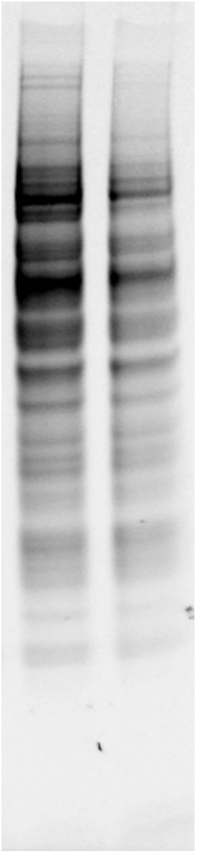

Figure 5c, glucose-dependence, HEK293, fluorescence

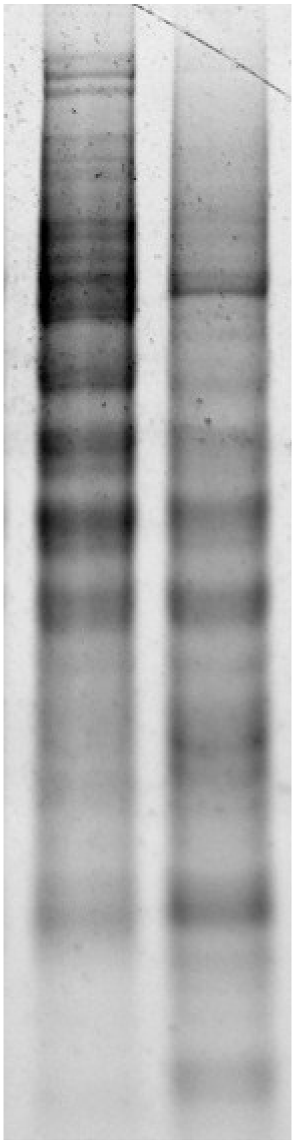

Figure 5d, p300i, fluorescence

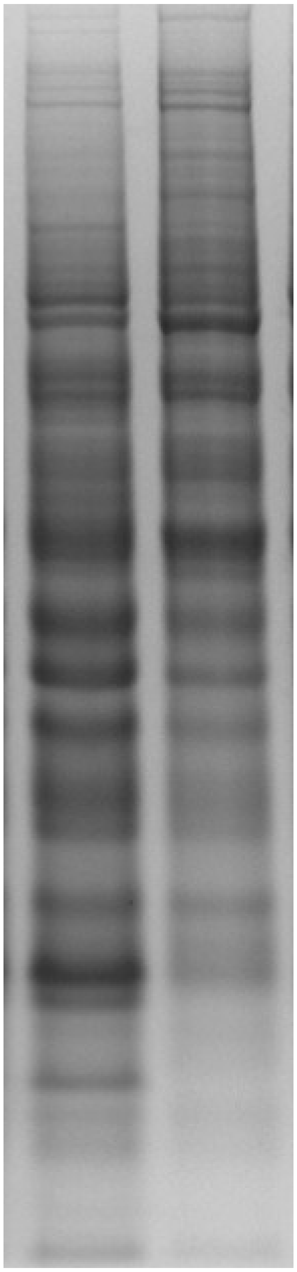

Figure 5d, p300i, Coomassie

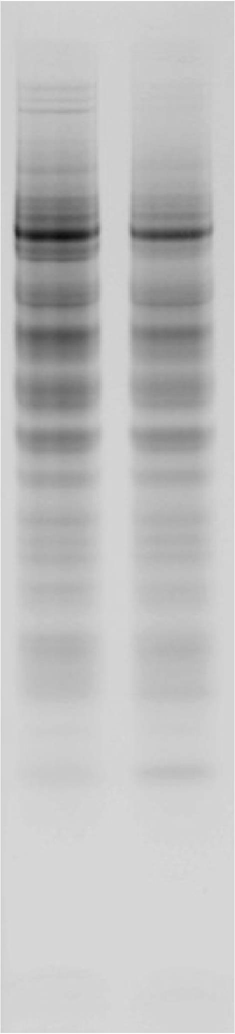

Figure 5e, HDACi, fluorescence

Figure 5e, HDACi, Coomassie

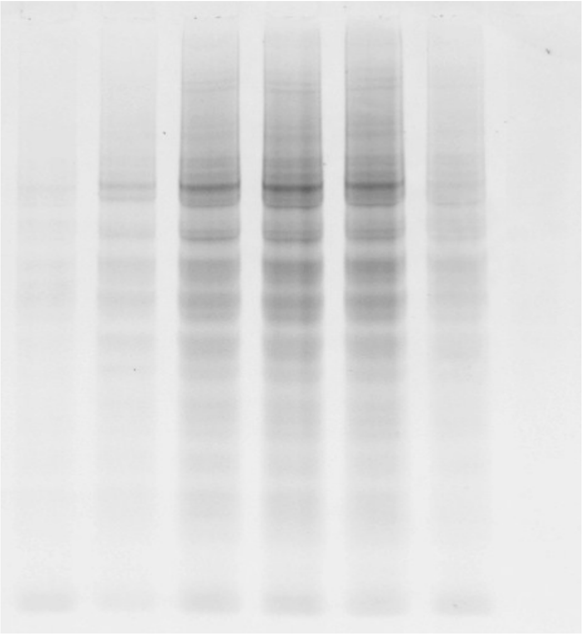

Figure S1d, time-dependence, fluorescence

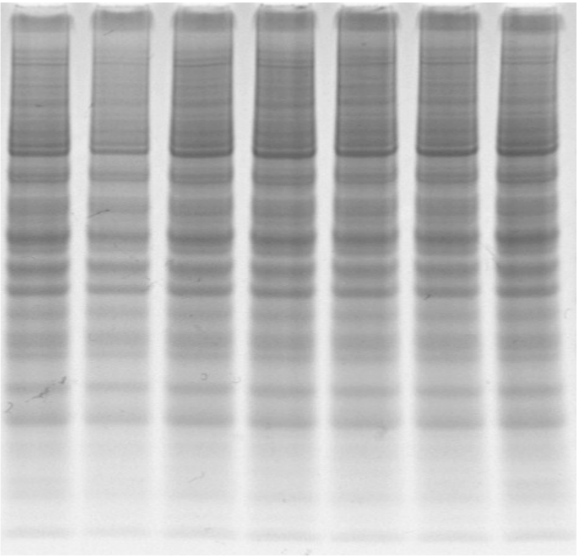

Figure S1d, time-dependence, Coomassie

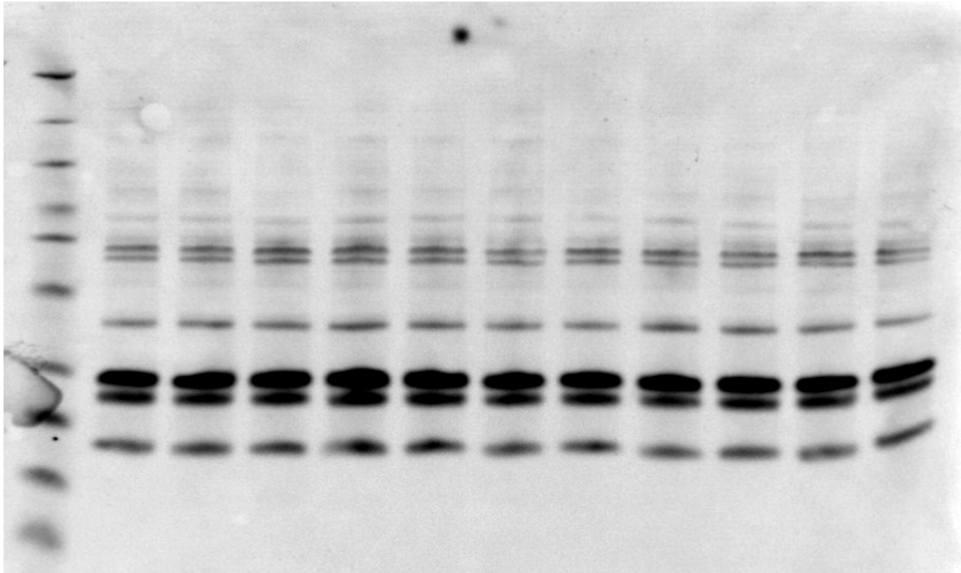

Figure S2a, Ponceau

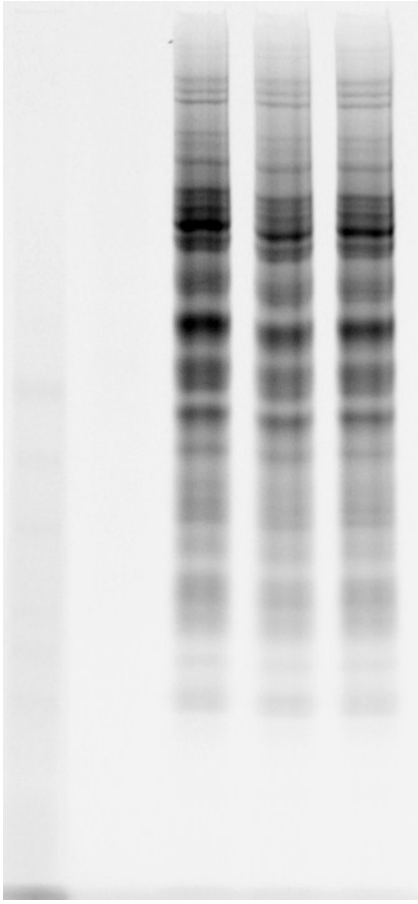

Figure S4, effect of FASNi on labeling, fluorescence

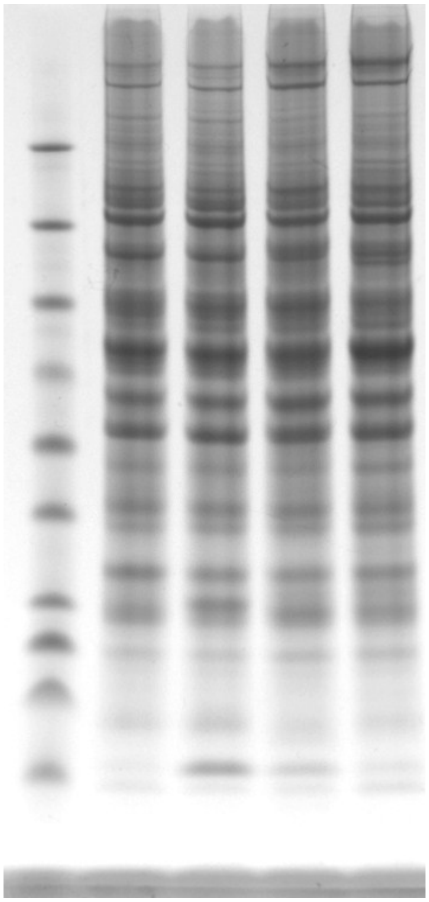

Figure S4, effect of FASNi on labeling, Coomassie

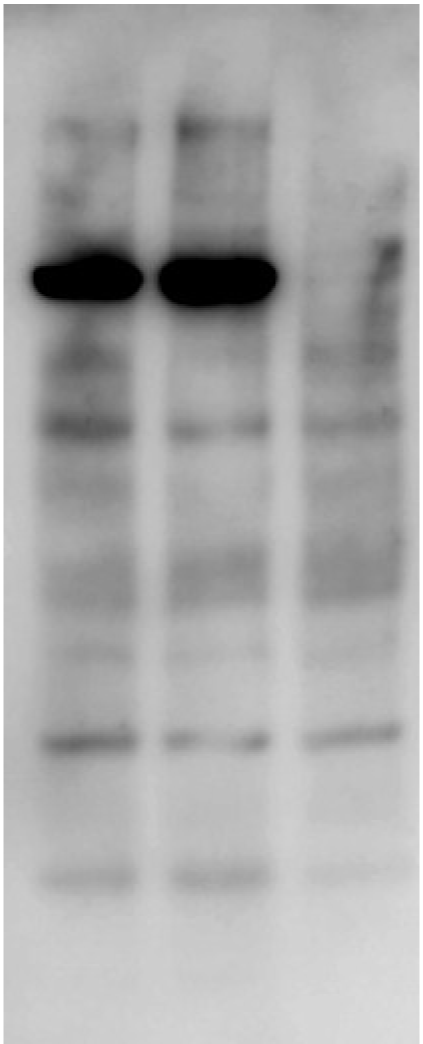

Figure S5, P300-FLAG overexpression, anti-FLAG immunoblot

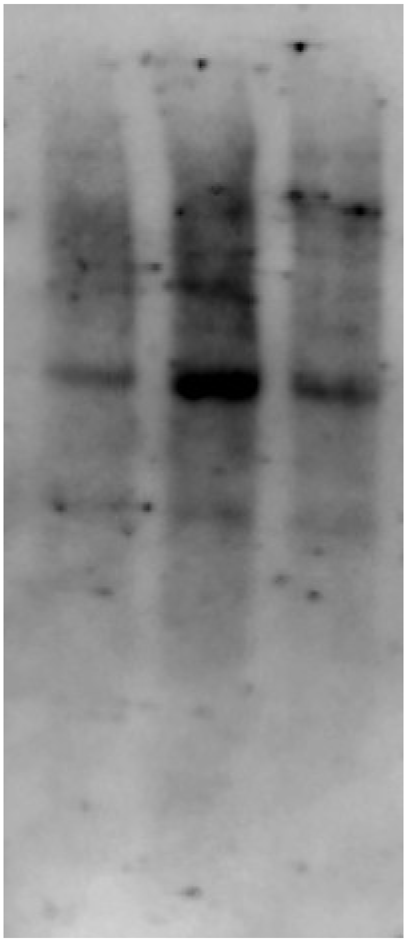

Figure S5, P300-FLAG overexpression, anti-biotin immunoblot.

References

1. *United States Pat.,* US20160235716 A1, 2015.
2. D. C. Montgomery and J. L. Meier, *Methods Enzymol*, 2016, **574**, 105-123.
3. D. C. Montgomery, A. W. Sorum and J. L. Meier, *Journal of the American Chemical Society*, 2014, **136**, 8669-8676.
4. G. Candiano, M. Bruschi, L. Musante, L. Santucci, G. M. Ghiggeri, B. Carnemolla, P. Orecchia, L. Zardi and P. G. Righetti, *Electrophoresis*, 2004, **25**, 1327-1333.
5. S. S. Basu and I. A. Blair, *Nat Protoc*, 2011, **7**, 1-12.
6. A. J. Worth, S. S. Basu, N. W. Snyder, C. Mesaros and I. A. Blair, *J Biol Chem*, 2014, **289**, 26895-26903.
7. E. M. Shonsey, J. Wheeler, M. Johnson, D. He, C. N. Falany, J. Falany and S. Barnes, *Methods Enzymol*, 2005, **400**, 360-373.
8. D. C. Montgomery, A. W. Sorum, L. Guasch, M. C. Nicklaus and J. L. Meier, *Chemistry & biology*, 2015, **22**, 1030-1039.
9. J. V. Lee, A. Carrer, S. Shah, N. W. Snyder, S. Wei, S. Venneti, A. J. Worth, Z. F. Yuan, H. W. Lim, S. Liu, E. Jackson, N. M. Aiello, N. B. Haas, T. R. Rebbeck, A. Judkins, K. J. Won, L. A. Chodosh, B. A. Garcia, B. Z. Stanger, M. D. Feldman, I. A. Blair and K. E. Wellen, *Cell Metab*, 2014, **20**, 306-319.
10. V. Vichai and K. Kirtikara, *Nat Protoc*, 2006, **1**, 1112-1116.
11. E. Weerapana, A. E. Speers and B. F. Cravatt, *Nat Protoc*, 2007, **2**, 1414-1425.
12. A. J. Frey, D. R. Feldman, S. Trefely, A. J. Worth, S. S. Basu and N. W. Snyder, *Anal Bioanal Chem*, 2016, **408**, 3651-3658.
13. S. S. Basu, C. Mesaros, S. L. Gelhaus and I. A. Blair, *Anal Chem*, 2011, **83**, 1363-1369.
